# Socio-economic factors as indicators for various animal diseases in Sardinia

**DOI:** 10.1371/journal.pone.0217367

**Published:** 2019-06-03

**Authors:** Federica Loi, Alberto Laddomada, Annamaria Coccollone, Elena Marrocu, Toni Piseddu, Giovanna Masala, Ennio Bandino, Stefano Cappai, Sandro Rolesu

**Affiliations:** 1 OEVR—Osservatorio Epidemiologico Veterinario Regionale della Sardegna, Cagliari, Italy; 2 Istituto Zooprofilattico Sperimentale della Sardegna “G. Pegreffi”, Sassari, Italy; 3 CeNRE—Centro Nazionale di Referenza per l’Echinococcosi/Idatidosi, Sassari, Italy; Panstwowy Instytut Weterynaryjny - Panstwowy Instytut Badawczy w Pulawach, POLAND

## Abstract

The need to consider the role of social factors in the efficacy of farm management and, consequently, in the onset and persistence of diseases typical to animal farms is increasingly being realized increasingly worldwide. Many risk analysis studies have been conducted to assess the role of various factors in the development of animal diseases; however, very few have accounted for the role of social factors. The aim of this work was to bridge this gap, with the main hypothesis that different socio-economic factors could be valid indicators for the occurrence of different animal diseases. A socio-economic analysis was performed using demographic characteristics of the farmers and data from 44 social indicators released by the Italian Statistician National Institute of Statistics (ISTAT) database. African swine fever (ASF) in wild boars (WB) and domestic pigs and other endemic animal diseases and zoonoses in Sardinia were considered, such as cistic echinococcosis (CE), contagious agalactia (CA), trichinellosis, West Nile disease (WND), and bluetongue (BT). Seven different negative binomial regression models were fitted using the number of cases between 2011–2017. Three indicators—cultural demand, employment rate, and legality—showed a statistically significant association with risk for all the diseases considered, but with varying effects. Some indicators, such as the age and sex of the farmer, material deprivation index, number of farms and animals, micro-criminality index, and rate of reported thefts were common to ASF, CA, trichinellosis, and CE cases. Others such as the forest surface and the energy produced from renewable sources were common to BT, WND, and ASF in WB. Tourism in seasons other than summer was a valid predictor of ASF and trichinellosis, while out-of-region hospital use had a statistically significant role in CE risk identification. These results may help understand the social context in which these diseases may occur and thus guide the design and implementation of additional risk management measures that go beyond well-known veterinary measures.

## Introduction

The One Health approach addresses the fundamental complexity and the relations that exist between human, animal, and ecological health. Recent conceptual developments have suggested that “all entities, including people, genes, animals, geologies, chemicals, and so forth, are potentially active in the development of a healthy public” [1]. Hidano et al. in 2018 accurately described the relationship between disease prevalence and farmer behaviours, which were influenced by various characteristics relating to the farms and farmers themselves (e.g., demographic factors, socio-economic status, and social network with other stakeholders). Since livestock populations are managed by humans, it is not surprising that culture and behaviour can influence the epidemiology of animal diseases [2]. The double relation between disease knowledge, economic situation and persistence of disease could be deducted by the fact that if farmers do not directly benefit financially from improving biosecurity, they will not invest in it [3] [4]. Livestock disease risk can be correlated with the socio-economic status of the farmer [5] [6] [7]. Disease prevention requires a comprehensive knowledge of the epidemiological context and the relevant risk factors/indicators, including health protocols as well as housing and management practices that impact on the social dynamics of farms [8]. Indeed, recent studies have suggested that each farmer develops different management and control strategies based on their social and cultural situation and disease understanding [9] [10]. As such, in this context, farmer information and implementation of adequate health education plans play a key role in disease management. Therefore, the complex relationship between disease spread and dynamic human behaviour needs to be considered for in disease simulation models to minimise potentially biased inferences [11]. Though, most studies have linked livestock health to individual animal health and management practices, they not account for human-related factors, as contemporary sociology has been shaped more by aspects of agricultural sustainability [12]. As early as 2009, Enticott underlined the importance of sociology in influencing the behaviour of all stakeholders involved in animal breeding, including farmers, and the impact of social science research in relation to the issue concerning animal disease [13]. An in-depth study of the association between animal disease and socio-economic status is even more important in areas that are defined as rural or developing regions [14] [15]. The distribution of foot-and-mouth disease in the world closely follows poverty indicators [14]. Collection of all different types of information related to ecosystems, animals, and human beings in a multidisciplinary view is also part of the concept of the American Veterinary Medical Association's One Health approach [16]. Many recent studies, focus on One health approach take into account social determinants to describe infectious disease spread and persistence [16] [17] [18] [19] [20]. Djordjevic et al. in 2003 described the re-emergence of trichinellosis in Serbia [21], and the report by Pozio et al. in 2006 described the first case of trichinellosis in Sardinia [22]. The authors affirmed that the association between human social conditions, illegal free-ranging pigs, and disease spread was very close and an in-depth study of human consumers was imperative. This strong association between culture, habits in animal breading and disease spread is all the more obvious regarding African Swine Fever (ASF). Studies in resource-poor pig-keeping communities have demonstrated that socio-economic factors are major constraints for control of ASF [3] [23]. The role of human population characteristics in the probability of West Nile virus (WNV) infection was confirmed a few years later by Ozdenerol et al. in 2008 [24], Liu in 2011 [25], and Gibney in 2012 [26]. In 2011, Drewe et al., found that social behaviours were implicated in the spread of bovine tuberculosis within a population [27], and Patterson et al., confirmed this association in 2017 [28]. Few studies take into account the role of social factors in the spread of contagious agalactia (CA): the first scientific review performed by Bergonier et al., in 1997, highlighted the importance of breeding conditions [29], while Madanat et al. in 2001 evaluated the differences in disease control measures between developed and developing countries [30]. However, to date, few studies correlate socio-economic factors and the development of livestock diseases, considering these social conditions and management of farms. Furthermore, these studies do not explore the associations in the context of the diseases included in the present study, with a particular focus on socio-economic factors. Sardinia is, arguably, an ideal place for this type of study as the region contains very rural areas, where several animal diseases are known to develop under seasonal influences. Several previous studies conducted in Sardinia have attempted to analyse the association between social factors and the development of different diseases; Firinu et al. in 1988 investigated the socio-economic factors which could influence the development of ASF, and described the fundamental role played by the type of farm and its management [5]. This hypothesis has subsequently studied in-depth by numerous studies [6] [7] [31] [32]. Furthermore, the spread of CE in association with social factors has also been studied in Sardinia, first by Brundu et al. in 2014 [33] and subsequently by Piseddu et al. in 2017 [34] and Loi et al. in 2019 [35]. Recently, following the succession of numerous seasonal epidemics of bluetongue (BT) in Sardinia, the role of social factors and farm management has been studied extensively, demonstrating the importance of good management practices in disease prevention and relaying information about risk factors to the farmers [36] [37]. Failure to consider health problems using an inter-disciplinary approach and to account for these dynamics may introduce a substantial layer of bias in human and animal health programmes [38] [39]. The underlying assumption of this work is that the social and economic conditions of an area, described by social ISTAT indicators, the material deprivation index (MDI) and the demographic features, could help to identify specific high-risk areas where each disease could spread in livestock. The aim of this work was to analyse the possible common role of social factors which characterise the human population, as risk indicators for animal diseases in Sardinia. The diseases considered were ASF in wild boar (WB) and domestic pigs (DP), WND in humans and animals, CE in sheep farms, BT in sheep and goats, and trichinellosis and CA in sheep and goats. The information that will arise from this work could be useful for stakeholders and should be considered in for disease control planning, in order to put into place targeted and specific disease control programmes for each territory.

## Material and methods

### Sardinian study context

Sardinia is an island with an area of 24,000 km^2^, located in the heart of the Mediterranean Sea (40°03′N 9°05′E) with a long history, comprising of a breath-taking countryside, miles of forest, pristine seas, old traditions, and largely uninhabited areas. For this reason, Sardinia has been defined as a micro-continent. Administratively (Regional Law 4^th^ February 2016), the region is divided into five provinces: Nuoro, Sassari, Oristano, South of Sardinia, and Cagliari (metropolitan city), with 377 municipalities. Although Sardinia is the third largest Italian region, in terms of area, it has the third lowest population density [40]. The low population density (69 inh./km^2^) and the presence of unpolluted areas make Sardinia a perfect region for a predominantly agro-pastoral culture, especially sheep and pig breading, which dates back to the Neolithic period (6^th^ century B.C.). Historically, Sardinia has been described by renowned writers as an ancient land, with habits different than those of the rest of the nation, and many places have been defined as rural [41]. These rural areas are still present in Sardinia, along with its ancient breeding and agricultural traditions; in 2000 the Sardinian region established the Rural Development Plan (http://www.regione.sardegna.it/documenti/1_43_20050210114924.pdf), which provides the list of municipalities defined as rural/deprived areas. Approximately 34% of Sardinian is used for agriculture and 60% for farming; the remainder is occupied by tree farming [42]. Sheep and goat farming have always been Sardinia’s primary economic source, while pig farming has generally been oriented toward individual family production and self-consumption [43] [44] [45]. Indeed, the culture of breeding one or a few pigs is still a very common practice, mostly in concomitance with sheep breeding [6] [7] [32] [46].

### Data collection

For the purposes of this work, an *ad hoc* database was created, collecting data from various sources, and considering a study period between 2011 and 2017. The principal analyses were performed considering all 377 Sardinian municipalities as an epidemiological unit arranged by study year with all variables were collected (S1 Table), from the relevant source. In order to pursue the main objective, data about farm owner characteristics, such as age, sex, level of education, type of farm, and socio-economic factors were matched with the numbers of cases for each disease considered in the present work. Case summaries for each disease were retrieved from the following main databases: Italian Veterinarian National Database (BDN), Veterinary Information Systems of the Italian Ministry of Health (VETINFO), Italian National Informative System for Animal Disease Notification (SIMAN), and Zoonosis National Information System (SINZOO). Specific data collection on slaughterhouses was performed by specific project of istituto Zooprofilattico Sperimentale della Sardegna (Italian Ministry of Health Current Research IZS SA 05/16RC) in order to collect data about CE-positive sheep. Farm owner characteristics were retrieved from the Italian National Institute of Statistics (ISTAT) database, specifically from AgriISTAT [47] and the socio-economic factors that were included were the MDI and Territorial Indicators for development policies released by ISTAT [48].

### Endpoint indicators

The outcome was reported as continuous variables and referred to the number of outbreak events for the year, for each disease considered, i.e, CE, ASF, WND, BT, trichinellosis, and CA. Based on official national data, an outbreak (i.e., case) was defined as a diagnosed disease event in accordance with the World Organization for Animal Health (OIE) Manual of Diagnostic Tests [49] and recorded in the SIMAN database. For each disease, an extraction query was used to collect information regarding province and municipality of the case, data of suspect diagnosis, data of diagnosis confirmation, and species. Specificity was required for CE cases as the data collection had been performed at the slaughterhouse level; due to the large number of sheep on the island, and assuming that a single CE-positive sheep was indicative of a positive farm, the number of positive sheep by municipality was used as the outcome. Table 1 shows the disease case distribution between 2011 and 2017. Since the outcome was the summary of cases for each disease, the dependent variables were derived from count data, following a count-data distribution (i.e., Poisson or negative binomial) rather than a normal distribution.

**Table 1:** Sardinian cases distribution, by study-year (2011–2017) and type of disease. The number of CE cases expressed as number of sheeps found positive during slaughterhouse inspection.

Year	Bluetongue	ASF (wild boar)	ASF (domestic pigs)	West Nile (human and animals)	CE (sheeps)	Trichinellosis	Contagious Agalactia (sheep and goats)
2011	6	2	33	52	844	10	59
2012	425	16	75	19	1088	5	67
2013	5756	67	109	5	754	2	83
2014	19	69	40	1	433	3	37
2015	16	79	16	5	396	0	60
2016	15	164	23	6	341	4	74
2017	2460	102	17	12	295	2	52
**Total**	**8697**	**499**	**313**	**100**	**4151**	**26**	**432**

### ISTAT indicators for developmental polices and socio-economic factors

The objective of this work was to identify as precisely as possible the different areas (municipalities), based on socio-economic and cultural conditions that could be indicative of material deprivation and evaluate their association with the presence of diseases in farms. For example, it would be somewhat obvious how the poverty rate could lead to material deprivation, in terms of dirty water or poor nutrition, which would be enough to describe a disadvantage social and cultural status [50]. In order to compose an accurate picture of the Sardinian farm context, specific covariates related to the socio-economic status, demographic characteristics of the farmers and the farms themselves have been collected by ISTAT and BDN. The choice of the ISTAT variable as indicators of material, social and cultural deprivation status was based on the World Heatlh Organization (WHO) publication, which summarised the main social determinants of health and well-being [51]. The ISTAT territorial indicators for development policies includes all the categories indicated by the WHO and focused on the relation between health and social status in the mail social studies [17] [19] [52] [53] [54] [55] [56]. In this way, the generalisability of the results can be ensured as much as possible. Furthermore, the association between CE and ISTAT territorial indicators for development policies has been successfully studied by Loi et al. in 2019 [35], providing interesting results and underling the need of in deep studies. Data on the sex and age of the farmers were collected using the fiscal code downloaded by BDN. The MDI was included in the model to explain the relation between human conditions and disease spread, and was defined as the “lack of goods, services, amenities, and physical environment” [57]. Cappai et al. in 2018, demonstrated the possible role of MDI as a valid indicator for ASF prediction [7]. The construction of the aggregate indices was carried out following the MDI methodology used by Cadum et al. in 1999, which was performed by ISTAT and the Università di Cagliari [48] [58]. The MDI was used as score and was represented by five rank quintiles (MDI-levels), following the example set by previous studies. The territorial indicators for development policies database contains 316 indicators available at the regional and/or sub-regional level, for macro-areas and the target areas of developmental policies. In the most cases, the time series began from 1995 and continued until the last available year. It is possible to consult the indicators, as well as the related data used to construct them, for thematic areas and reference areas of planned developmental policies. The database is updated monthly, around the 20^th^ of each month, except in August. As described in the explanatory note updated monthly (http://www.istat.it/storage/politiche-sviluppo/nota_esplicativa.pdf), “The database is one of the products envisaged by the Disciplinary Agreement between ISTAT and the Department for Economic Development and Cohesion (DPS)”, within the project "Sectoral Territorial Statistical Information for Structural Policies 2010–2015" funded by PON Governance and ERDF technical assistance. The choice of using these indicators to explain a phenomenon related to the social conditions of the population started from the fact that these are the most updated and complete databases produced by ISTAT. In fact, this database constitution arises from the need to have statistical information disaggregated at the territorial level, growing in relation to the support they offer to public decision makers for planning and evaluation of structural and development policies. As not all indicators were available at the same territorial level, only those released at the municipality level were considered for this study.

### Statistical analyses

Data quality was assessed in terms of accuracy, completeness, and missing information. Descriptive analyses were performed to evaluate the baseline distribution of each included variable, and to select the most appropriate ones. Table 2 shows the baseline distribution for each variable collected, both overall and by province. Most variables collected were quantitative and are expressed as mean values, standard deviations (SD), median, and interquartile range (IQR). Categorical variables were described by frequencies and percentages. The variables reported in S1 Table were evaluated as potential covariates in our modelling analyses, considering both experimental and statistical requirements (e.g. non-collinearity). When a linear relationship was assumed, bivariate analyses was performed to evaluate its strength, by means of the Spearman non-parametric correlation coefficient to avoid collinearity, which can lead to wrong parameter estimation. Variables with correlation coefficient greater than 0.8 were excluded from the analysis [59]. Initially each of the factors listed above were tested in a univariable negative binomial regression model. Variables that were significant in the univariable model (p ≤ 0.20) were considered for inclusion in the multivariable model. A step-wise selection process was used to include factors into the multivariable model based on a likelihood ratio test [60]. Multicollinearity between variables was tested because the estimation remained unbiased, even if ordinary least squares assumptions were not violated [61] [62] [63]. Since a bivariate relationship between two predictors is not sufficient to diagnose the presence of multicollinearity, the method suggested by Yu et al. in 2015 [64], based on a variance inflation factor (VIF), was used to identify and eliminate potentially redundant features [65]. A VIF value > 1 indicated the risk of multicollinearity, while values > 10 indicated serious multicollinearity problems. If multicollinearity was detected, the responsible predictors were identified and one or more of the highly correlated predictors were removed. Although the count data often follow a Poisson distribution, they were tested with the Likelihood ratio test for alpha parameters, demonstrating evident overdispersion in each number of disease cases [66]. Due to overdispersion and the presence of excessive zero values (83%), the models that best fit the data were the negative binomial regression models (NBRM). Negative binomial regression is a type of generalised linear model in which the dependent variable Y is the total number of events occurring in a specific space-time interval. A convenient parameterisation of the negative binomial distribution given by Hilbe in 2011 [67] and by Rabe-Hesketh and Skrondal in 2012 [68] is derived from a Poisson-gamma mixture, or alternatively, the number of failures before the (1/α)^th^ success. Assuming that the observations between years and municipalities were not independent, a mixed-effects NBRMs was applied (Eq (1)), including ‘year’ and ‘municipality’ as random effects. The inclusion of ‘years’ and ‘municipality’ as random effects made it possible to control the ‘between-year’ and ‘between-municipality’ differences in the model and to take into account the dependency or correlation of observations. Using the method proposed by Hocking in 1976 [69], random effects were included in the model once the model with the best fit was selected based on an adjusted R^2^, Bayesian information criterion (BIC) [70] and Akaike's information criterion (AIC) [71] values. Considering a series of M independent clusters, and conditional on the latent variable *ζ*_*ij*_ and a set of random effects **u**_*j*_,

$$y_{ij}|\zeta_{ij}∼Poisson\;(\zeta_{ij})$$

and

$$\zeta_{ij}|u_{j}∼Gamma\;(r_{ij},p_{ij})$$

and

$$u_{j}∼N\;(0,\Sigma )$$

**Table 2:** Descriptive analysis of baseline variables collected, by Sardinian provinces (n = number of municipalities), observed from 2011 to 2017. Data are presented as mean (SD); median [IQR], calculated by year in study.

VARIABLE COLLECTED	Cagliari	Sassari	Nuoro	Oristano	Sud Sardegna
**N. farms,** by species					
** Sheep and goats**	2994 (233)	3254 (302)	3312 (405)	2437 (156)	3709 (147)
** Bovine**	818 (74)	1952 (125)	1719 (276)	1437 (196)	1023 (98)
** Swine**	2541 (361)	2842 (487)	2361 (316)	3112 (275)	3492 (375)
** Horse**	1584 (217)	2270 (389)	1505 (186)	1526 (144)	2070 (402)
**N. animals,** by species					
** Sheep and goats**	650757 (9127)	878401 (10486)	861780 (10365)	574762 (9567)	927457 (10678)
** Bovine**	22099 (3215)	49175 (5985)	57710 (6231)	63823 (6528)	29514 (2988)
** Swine**	42922 (5781)	28438 (2455)	18654 (1988)	31226 (2561)	7744 (5477)
** Horse**	9613 (255)	39784 (5127)	28361 (4126)	29858 (7452)	75919 (10154)
**% of female farms owner**	22 (3.7); 21.6 [19.5–25.7]	24 (1.5); 24.6 [22.8–25.7]	26 (1.9); 25.8 [24.2–27.5]	28 (1.3); 28.6 [26.9–29.1]	25 (1.7); 24.9 [22.9–26.1]
**Age of farmer**	52.4 (2.2); 52 [50-55]	48.1 (1.8); 48 [47-50]	56.6 (1.5); 56 [55-58]	57.2 (1.8); 57 [56-59]	55.5 (1.9); 55 [54-57]
**IDM quintile**					
** 1 (very wealthy)**	4 (23.5%)	17 (18.5%)	12 (16.2%)	6 (6.9%)	23 (21.5%)
** 2 (wealthy)**	2 (11.8%)	12 (13%)	10 (13.5%)	18 (20.7%)	16 (15.0%)
** 3 (medium)**	6 (35.3%)	22 (23.9%)	18 (24.3%)	27 (31.0%)	21 (19.6%)
** 4 (deprived)**	2 (11.8%)	13 (14.1%)	13 (17.6%)	19 (21.8%)	12 (11.2%)
** 5 (very deprived)**	3 (17.6%)	28 (30.4%)	21 (28.4%)	17 (19.5%)	35 (32.7%)
**Ind_007—Unpolluted coasts for pollution**	2.5 (0.05); 2.5 [2.4–2.55]	4.6 (0.07); 4.7 [4.5–4.7]	2.0 (0.06); 2.0 [1.9–2.1]	5.0 (0.1); 5.1 [5.0–5.2]	1.8 (0.1); 1.9 [1.8–2.0]
**Ind_009 –Efficiency of water distribution**	53.7 (1.2); 54 [51-55]	53.6 (2.1); 53 [51-56]	56.9 (1.9); 57 [55-59]	(1.6), [55 [52-57]	53.1 (1.4); 53 [51-52]
**Ind_012—Unemployment rate**	15 (2.8); 15.5 [12.5–17.7]	16.8 (2); 16.7 [15.9–18.7]	11 (1.9); 10 [9.9–11.7]	17 (2); 17.4 [15.1–19.7]	18 (2.9); 18.6 [15.7–21.2]
**Ind_013—Employment rate**	51.7 (1.35); 52 [50.4–52.7]	50.1 (2.0); 51.2 [47.5–51.7]	51.3 (1.6); 50.7 [50.5–52.8]	50.1 (1.2); 50.4 [49-51]	49.8 (1.5); 49.9 [47.6–52.1]
**Ind_014—Employment rate over 54 years**	42.6 (7.2); 41 [38.5–45.9]	39.7 (8.5); 42.0 [39.8–45.2]	39.5 (7.9); 40.8 [31.3–43.1]	38.0 (4.8); 38.4 [35.8–45.9]	33.0 (7.5); 29.9 [27.4–37.7]
**Ind_015 –Young unemployment rate**	35.4 (6.8); 34.4 [29.4–41]	53.5 (4.9); 52.7 [49.6–57.5]	55.2 (14); 55.6 [42.5–68.1]	51.5 (11); 52 [44.9–59.6]	1.4 (8.5); 47.3 [42.8–53.5]
**Ind_018—Cultural demand**	21.8 (7.9); 18.2 [17.6–28.4]	9.3 (0.85); 9.3 [8.7–10.3]	3.8 (2.4); 2.9 [2.7–3.4]	24.6 (11.5); 23.7 [10.8–36.1]	7.2 (3.1); 7.5 [6.8–8.5]
**Ind_024—Degree of promotion of the cultural offer of state institutions**	308 (420); 198 [67–236]	45 (23); 38 [31-41]	73 (75); 40 [35-70]	838 (66); 941 [194–1232]	47 (19); 45 [36–48]
**Ind_027—Degree of diffusion of theatrical and musical entertainment**	58.0 (11.6); 52.1 [51.3–67.3]	45.2 (5.3); 44.4 [42.3–49.4]	23.2 (5.0); 22.9 [20.3–25.3]	25.3 (2.9); 26.5 [23.9–26.7]	39.6 (15.6); 38.6 [26.4–52.3]
**Ind_044—Air traffic index**	1222 (116); 1228 [1085–1615]	641 (23); 646 [621–661]	465 (25); 461 [459–488]	7.2 (10); 0 [0–19]	602 (110); 602 [589–618]
**Ind_052—Separate municipal waste**	49.5 (2.4); 49.5 [46.6–52.1]	43.6 (5.6); 44 [37.7–49.4]	54.2 (5.9); 56.1 [49.9–60]	62.5 (2.3); 63 [60.9–64.9]	59.5 (6.3); 60.5 [59.1–62.4]
**Ind_057—Difference between male and female employment rate**	17.2 (1.9); 16.8 [15.1–18.3]	14.9 (2.5); 15.2 [11.9–17.1]	14.8 (3.9); 16.1 [12.3–18.5]	17.5 (2.3); 18.3 [16.2–19.1]	23.7 (6.6), 22.8 [18.8–27.6]
**Ind_060 –Interruption of the electricity service**	3.6 (0.63); 3.5 [3.2–3.8]	2.3 (0.29); 2.3 [2–2.5]	2.5 (0.47); 2.5 [2.3–2.8]	4.02 (1.4); 3.8 [3.1–4.1]	3.5 (1.2); 3.3 [2.5–4.1]
**Ind_080—Energy produced from renewable sources**	2.8 (0.92); 2.7 [2.5–3.2]	7.2 (2.1); 6.7 [5.7–7.8]	18.9 (10.2); 13.7 [10.9–29.1]	8.0 (3.1); 6.8 [6.0–8.2]	5.9 (3.9); 5.8 [4.7–5.3]
**Ind_083—Municipal waste**	466 (26.6); 458 [438–492]	452 (28.4); 381 [368–394]	356 (28.9); 348 [329–379]	381 (12.3); 381 [369–393]	408 (22.1); 417.8 [389–420]
**Ind_105—Tourism rate**	4.9 (0.43); 4.8 [4.6–5.3]	5 (0.48); 4.9 [4.7–5.2]	6.4 (0.93); 6.6 [5.9–7.2]	2.8 (0.33); 2.6 [2.5–3.0]	3.5 (0.55); 3.8 [2.1–4.5]
**Ind_108—Participation of the population in the labour market**	61.1 (1.6); 60.3 [60–62.5]	60.4 (1.5); 59.6 [59.4–61.7]	57.8 (1.5); 57.7 [56.3–58.2]	60.5 (1.9); 61.2 [58.6–62.3]	55.7 (2.5); 56.1 [54.4–57.2]
**Ind_120—Weight of cooperative society**	6.9 (0.58); 6.8 [6.4–7.7]	2.5 (0.25); 2.5 [2.4–2.8]	3.3 (0.32); 3.2 [3.07–3.7]	9.2 (0.78); 8.9 [8.7–9.5]	2.1 (0.9), 2.1 [1.3–2.9]
**Ind_135—Micro criminality index**	5.3 (0.32); 5.2 [5.1–5.6]	4.1 (0.33); 4.0 [3.9–4.5]	3.0 (0.52); 2.9 [2.4–3.6]	5.0 (0.76); 4.9 [4.5–5.3]	4.7 (0.45); 4.7 [4.4–4.8]
**Ind_141—Hospital emigration**	4 (0.24); 4 [3.8–4.3]	4.8 (0.17); 4.9 [4.7–5]	5.7 (0.56); 5.5 [5.2–6.3]	4.9 (0.42); 5.1 [4.4–5.3]	3.7 (1.1); 3.5 [2.9–4.3]
**Ind_142—Childhood services**	50 (5); 50.7 [45.1–51]	31.4 (3.9); 28.8 [27.8–36.4]	35.7 (3.8); 38.5 [32.7–38.5]	22 (2.1); 22.7 [20.5–23]	37.8 (3.8); 39.5 [35.4–40]
**Ind_162—Funding risk**	3.5 (1.1); 3.1 [2.4–4.6]	4.3 (2.2); 3.1 [2.7–6.6]	4.3 (1.3); 4.8 [2.8–5.4[	2.9 (0.96); 2.5 [2.4–2.8]	3.7 (2.8); 2.8 [1.7–4.9]
**Ind_165—Tourism in not-summer period**	0.98 (0.13); 0.94 [0.91–1.13]	1.03 (0.09); 1.01 [0.97–1.11]	0.92 (0.25); 0.87 [0.75–1.14]	0.71 (0.076); 0.71 [0.65–0.75]	0.75 (0.15); 0.76 [0.65–0.78]
**Ind_168—Export index**	4.8 (0.85); 5 [4.4–5.2]	30 (7.5), 26 [23–36]	37 (24); 45 [12–59]	6.8 (4.1); 6.4 [5.7–7.8]	5.7 (3.5); 5.5 [4.8–7.2]
**Ind_175—Unemployment rate (male)**	14.5 (3.64); 13.7 [11.7–13.6]	16.8 (3.02); 16.5 [15.4–13.5]	10.8 (2.13); 10.6 [9.3–13.2]	15.2 (3.44); 14.7 [13.7–13.9]	16.8 (7.42); 13.8 [11.5–14.0]
**Ind_176—Unemployment rate (female)**	14.6 (1.90); 13.9 [13.1–15.3]	18.2 (2.79); 16.9 [16.6–20.7]	12.3 (2.49); 12.2 [10.8–14.1]	18.7 (2.9); 19.7 [16.7–20.8]	22.0 (2.41); 20.1 [19.3–20.6]
**Ind_177—Employment rate (male)**	61.3 (2.59): 60.7 [59.7–62.9]	57.0 (2.64); 57.3 [55.3–59.9]	59.1 (3.12); 59.7 [57.3–61.9]	59.3 (2.15); 58.6 [57.8–60.2]	56.7 (5.59); 58.2 [54.0–61.1]
**Ind_178—Employment rate (female)**	43.4 (1.28); 43.9 [42.9–44.4]	41.9 (2.85); 42.7 [39.9–43.3]	43.5 (1.92); 43.8 [43.2–45.2]	41.4 (2.24); 41.6 [39.9–42.4]	33.0 (2.06); 33.7 [31.7–34.2]
**Ind_232—Percentage of municipal waste disposed of in landfills**	45 (16); 33.5 [33–64.2]	90 (10.6); 95.8 [77.5–99.1]	60.5 (13); 69 [44.8–70]	214 (14.3); 224 [197.4–225]	29.7 (13.7); 26 [16.3–45.6]
**Ind_239—Forests surface**	3731 (2884); 2795 [1891–7806]	4087 (3160); 3062 [2071–8551]	4757 (3678); 3564 [2411–9952]	1076 (832); 806 [545–2251]	2081 (799); 1976 [799–2751]
**Ind_241—Enrolment gross rate in the business register**	6.4 (0.25); 6.4 [6.1–6.6]	6.3 (0.38); 6.3 [6.2–6.4]	5.9 (0.46); 6 [5.3–6.2]	6.15 (0.36); 6.2 [5.9–6.4]	5.9 (1.77); 5.9 [5.8–6]
**Ind_242—Enrolment rate in the business register**	0.52 (0.31); 0.54 [0.31–0.63]	0.82 (0.34); 0.96 [0.44–1.1]	1.3 (2.03); 0.51 [0.20–1.2]	0.88 (0.34); 0.95 [0.49–1.2]	0.79 (0.33); 0.81 [0.52–1.1]
**Ind_255—Forests surface burned by fire**	0.5 (0.48); 0.3 [0.21–0.65]	0.4 (0.36); 0.2 [0.13–0.53]	0.8 (0.69); 0.5 [0.29–0.90]	0.7 (0.63); 0.4 [0.31–0.92]	0.9 (0.12); 0.7 [0.45–0.98]
**Ind_265—Air quality monitoring**	3.4 (0.2); 3.4 [3.2–2.5]	3.2 (0.23); 3.3 [3–3.4]	3.5 (0.5); 3.2 [3.1–3.8]	1.9 (0.22); 1.8 [1.7–1.9]	3.3 (0.25); 3.2 [2.9–3.4]
**Ind_278—Flood risk population**	6.1 ab / km^2^	1.43 ab / km^2^	0.89 ab / km^2^	5.2 ab / km^2^	5.6 ab / km^2^
**Ind_279—Rate of reported thefts**	18.8 (1.7); 19.2 [17.5–24.7]	21.4 (2.3); 21.7 [20.8–24.9]	15.8 (2.1); 16.2 [14.6–24.7]	8.5 (1.17); 8.3 [7.8–24.7]	17.2 (1.9); 16.8 [15.4–25.4]
**Ind_280—Rate of reported robberies**	0.37 (0.05); 0.37 [0.32–0.42]	0.33 (0.04); 0.33 [0.29–0.37]	0.38 (0.12); 0.38 [0.37–0.46]	0.11 (0.02); 0.11 [0.08–0.12]	0.29 (0.07); 0.29 [0.25–0.47]
**Ind_281—Homicides rate**	0.9 (0.55); 0.89 [0.36–1.43]	1.2 (0.77); 0.91 [0.6–2.1]	5.1 (1.8); 5.6 [3.8–5.7]	1.04 (0.57); 1.21 [0.61–1.22]	1.07 (0.81); 0.73 [0.64–1.09]
**Ind_414—Taking charge of all users of childcare services**	13.6 (2); 13.1 [12.9–13.6]	16 (3.4); 14.5 [14–18.3]	15.2 (2.3); 14.3 [13.9–14.6]	11.6 (1.3); 10.8 [10–13.4]	12.6 (1.5); 11.8 [10.2–14.2]
**Ind_415—Elderly in social assistance**	1.9 (0.08); 1.9 [1.7–1.9]	2.25 (0.11); 2.2 [2.1–2.3]	2.9 (0.21); 2.8 [2.7–2.9]	3.68 (0.14); 3.7 [3.6–3.8]	3.89 (0.18); 4.1 [3.5–4.3]
**Ind_445—Index of accessibility to urban nodes**	77.5 (18); 72 [66.7–81.7]	65.4 (11.2); 67 [64.8–69.2]	58.1 (12); 62 [55 – 59]	70.6 (14); 65 [61 – 72]	120.4 (21); 116 [110–123]

Where *y*_*ij*_ is the count response of the *i*^th^ observation, *i* = 1, …, *n*_*j*_, from the *j*^th^ cluster, *j* = 1, …, *M*, and *r*_*ij*_ and *p*_*ij*_ were parameterised using the mean overdispersion:

$$r_{ij}=\frac{1}{\alpha }\;and\;p_{ij}=\frac{1}{1+{\alpha \mu }_{ij}}$$

The random effects **u**_*j*_ are *M* realisations from a multivariate normal distribution with a mean of 0 and *q×q* variance matrix **Σ**. The random effects are not directly estimated as model parameters but are instead summarised according to the unique elements of **Σ**, known as variance components. The probability that a random response *y*_*ij*_ takes the value *y* and can be modelled by Mixed-effects NBRM is then given by:

$$Pr(y_{ij}=y|u_{j})=\frac{\Gamma (y+r_{ij})}{\Gamma (y+1)\Gamma (r_{ij})}{p_{ij}}^{r_{ij}}{\left(1-p_{ij}\right)}^{y}$$ (1)

Seven different NBRMs were fitted using the number of cases of ASF in DP, ASF in WB, BT, CE, WN, trichinellosis, and CA, as the outcome. Each fitted Mixed-effect NBRM was compared with the corresponding Mixed-effect Poisson regression model using the Likelihood-ratio test [72]. The logistic multilevel mixed model results are presented as adjusted odds ratio (OR_adj_), which was calculated with the method proposed by Gardner in 1995 [73] and made it possible to account for the effect due to all the additional variables included in the analysis. A method to test for errors in models created by step-wise regression was to assess the model against a set of data that were not used to create the model [74]. This procedure was performed by building the Sardinian models based on a ‘training dataset’ (years 2011–2018) and using the data of the entire Italian nation (excluding Sardinia) during the same years (years 2011–2018) as a ‘test dataset’ to assess the accuracy of the model (external validation). This was possible for all the diseases considered, except for ASF and trichinellosis, since these diseases are still present only in Sardinia. The external validation for ASF and trichinellosis was performed using data between 2015 and 2018 as the ‘training dataset’ and retrospective data between 2011 and 2014 as ‘test dataset’. The Italian national data used as the ‘test dataset’ are summarised in S2 Table. The predicted performance of each Mixed-effect NBRM was measured by Spearman’s correlation coefficient between the predicted and observed values [75], and the goodness-of-fit was evaluated using the root mean square (Root MSE) test statistic and residual analysis [76]. Finally, in order to give a generic picture of the disease risk based on social condition, after the estimation of the singularly variables influence for any disease by statistical multivariable models, qualitative comparisons between predictive variables for each disease model were performed, comparing the features that were commonly associated to one or more diseases with the number of cases. All statistical analyses were performed using Stata 13 (StataCorp, Stata statistical software: Release 13, StataCorp LP, College Station, TX, USA [2013]) and R-opensource (Version 3.3.2, R-Foundation for Statistical Computing, Vienna, Austria), setting a statistically significant p-value of 0.05.

## Results

All 377 Sardinian municipalities were observed for 7 years (2011–2017), and at least 2,639 records were analysed. A total of 316 Territorial Indicators for development policies were collected, 151 of which were excluded from the analysis as they were unavailable at municipality level, and 124 Indicators were excluded due to multicollinearity problems. A total of 44 ISTAT Indicators were included. General data description collected from SIMAN, BDN, and ISTAT are reported in Table 2, categorised by province. The number of sheep farms was similar in all provinces, except Oristano, where it was lower. This was not surprising as, historically, Oristano has been more dedicated to bovine breeding, as can been observed by the number of cattle. The number of female farm owners was greater in the Nuoro and Oristano provinces, while a younger owner population was recorded in Cagliari and Sassari. Most of the municipalities in the Cagliari and Oristano provinces were at a medium condition of MDI (36% and 31%, respectively), while, in the other provinces, most of the municipalities existed in very deprived conditions. It is interesting to observe that the Oristano province had the lowest average surface area of burnt forest (median = 808, IQR [545–2251]), while the Cagliari province recorded the highest index of ‘at flood risk’ population (6.1 ab/km^2^). All variables related to cultural demand or cultural heritage were at their minimum in the Nuoro province and at their maximum in Oristano and Cagliari. Variables related to work seemed to be equally distributed all over Sardinia, with a slightly higher unemployment rate (11–17%) compared to the national average (10%) [40]. Data about legality and security showed variability across provinces; Sassari had a higher rate of reported thefts whereas Oristano had the lowest rate, homicides were more frequent in Nuoro compared to other provinces and, in general, a higher micro-criminality rate was reported in Cagliari. Health services showed a different but expected distribution, following the age distribution trend across the provinces; the oldest population was recorded in Oristano and Nuoro, where the proportion of elderly in social assistance was high (4% and 3%, respectively), while childcare services were more common in Cagliari and Sassari, where a younger population was more concentrated. The highest tourism rate was noted in Nuoro during all seasons (mean = 6.4, standard deviation (SD) = 0.93), and in Sassari during the non-summer period (mean = 1.03, SD = 0.09).

### NBRM for African swine fever in domestic pigs

**Fig 1:**
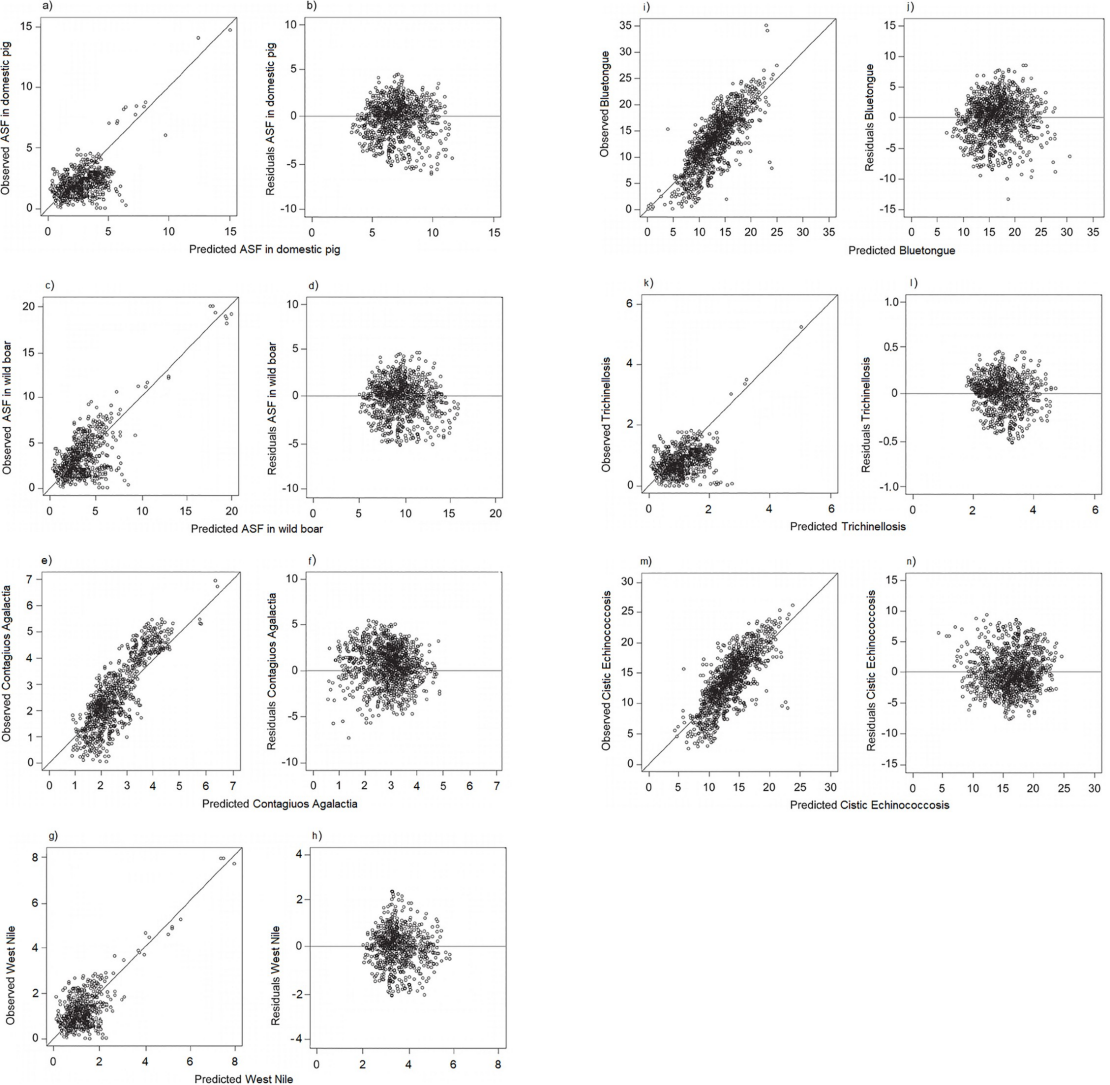
(a-n). Diagnostic plots of the seven prediction model. Graph (a, c, e, g, i, k, m) shows observed versus predicted values with a line that has zero intercept and slope equal to one. Graph (b, d, f, h, j, l, n) shows residuals versus predicted values. Predicted and residuals were calculated using only the fixed effects. The “hexbin” function in hexbin R-package has been used to separate the overlapped points.

Risk factors that had a p-value ≤ 0.20 in the univariable analysis are presented in S3 Table. A total of 27 variables related to ASF in DP between 2015–2018 have been considered for their inclusion into the final Mixed-effects NBRM; 14 of these were removed from the final Mixed-effect NBRM after ascertaining multicollinearity (statistically significant Spearman test and/or VIF > 10). The results obtained by the Mixed-effect NBRM are shown in Table 3 and expressed as the adjusted odds ratio and confidence interval at 95% probability (OR_adj_ [95% CI]), and the p-value. The number of farms in each municipality and the number of animals in the livestock were significant risk indicators, showing a tendency to favour an outbreak with an OR_adj_ of 1.08 at p = 0.005 ([95% CI = 1.02–1.11]) calculated per 10 farms, and an OR_adj_ of 1.01 at p < 0.0001 ([95% CI = 1.01–1.02]), per 100 animals. A greater age of the farmer seems to have a protective role against increasing the number of outbreaks (OR_adj_ = 0.80, [95% CI = 0.77–0.84], p < 0.0001), as does the female sex of the farmer compared to the male (OR_adj_ = 0.51, [95% CI = 0.33–0.82], p = 0.005). Comparing the first MDI level (lower deprivation) with others, the OR_adj_ suggested an increasing probability of ASF outbreak in farms, with statistically significant results between MDI level-1 and MDI level-4 (OR_adj_ = 2.23, [95% IC = 1.70–2.95], p < 0.0001), or MDI level-5 (OR_adj_ = 2.64, [95% CI = 1.90–3.69], p < 0.0001). With regards to the ISTAT indicators for sociology, the NBRM highlighted a low probability of outbreak in farms located within municipalities with high employment rates (Ind_013, OR_adj_ = 0.77, [95% CI = 0.62–0.96], p = 0.019), and with growing cultural demand (Ind_018, OR_adj_ = 0.95, [95% CI = 0.93–0.98], p = 0.001). All indicators related to criminality were able to identify municipalities at a higher ASF risk in Sardinia. The probability of observing an outbreak in each municipality increased by 46% (OR_adj_ = 1.46, [95% CI = 1.7–1.98], p = 0.016) with an increase of one point in the index of micro-criminality (Ind_135), or 58% (OR_adj_ = 1.58, [95% CI = 1.51–1.28], p < 0.0001) in the rate of reported thefts (Ind_279), and increased by 7% (OR_adj_ = 1.07, [95% CI = 1.03–1.13], p = 0.001) with an increase of one point in the rate of reported robberies (Ind_280). Higher counts in outcome variables were observed if the number of inhabitants at flood risk (Ind_278) was higher (OR_adj_ = 1.48, [95% CI = 1.11–1.99], p = 0.009) and counts doubled when the municipality was characterised by high levels of tourism in non-summer seasons (Ind_165) (OR_adj_ = 1.47, [95% CI = 1.31–1.65], p < 0.0001). The Likelihood-ratio test obtained (LR chi^2^ = 213.18, prob > chi^2^ = 0.0001), favoured the mixed-effect NBRM against the mixed-effect Poisson regression model. Data used for external validation (between 2011and 2014) are reported in Table 1. The predicted performance of the final regression model for ASF was tested by analysing the regression’s residuals, both within the “training dataset” (i.e. internal validation) and the “test dataset” (i.e. external validation). The model shown to be able to predict the correct outcome properly with a strong goodness-of-fit, according to internal validation criteria (residual mean = 3.22*10^−6^, SD = 1.03*10^−6^, Spearman’s correlation coefficient = 0.837, p < 0.0001) and external validation (residuals’ mean = 4.38*10^−3^, SD = 2.59*10^−3^, Spearman’s correlation coefficient = 0.849, p < 0.0001). The root mean square tests were insignificant for both datasets (Root MSE = 0.186, p-value = 0.76; Root MSE = 0.175, p-value = 0.84, respectively), indicating no evidence of failure. Fig 1A and 1B are a graphical representation of the model’s residuals and agreement between the observed values within the ‘test dataset’.

**Table 3:** Descriptive analysis of baseline variables collected, by Sardinian provinces (n = number of municipalities), observed from 2011 to 2017. Data are presented as mean (SD); median [IQR], calculated by year in study.

VARIABLE COLLECTED	Cagliari	Sassari	Nuoro	Oristano	Sud Sardegna
**N. farms by species**					
** Sheep and goats**	2994 (233)	3254 (302)	3312 (405)	2437 (156)	3709 (147)
** Bovine**	818 (74)	1952 (125)	1719 (276)	1437 (196)	1023 (98)
** Swine**	2541 (361)	2842 (487)	2361 (316)	3112 (275)	3492 (375)
** Horse**	1584 (217)	2270 (389)	1505 (186)	1526 (144)	2070 (402)
**N. animals by species**					
** Sheep and goats**	650757 (9127)	878401 (10486)	861780 (10365)	574762 (9567)	927457 (10678)
** Bovine**	22099 (3215)	49175 (5985)	57710 (6231)	63823 (6528)	29514 (2988)
** Swine**	42922 (5781)	28438 (2455)	18654 (1988)	31226 (2561)	7744 (5477)
** Horse**	9613 (255)	39784 (5127)	28361 (4126)	29858 (7452)	75919 (10154)
**% of female farms owner**	22 (3.7); 21.6 [19.5–25.7]	24 (1.5); 24.6 [22.8–25.7]	26 (1.9); 25.8 [24.2–27.5]	28 (1.3); 28.6 [26.9–29.1]	25 (1.7); 24.9 [22.9–26.1]
**Age of farmer**	52.4 (2.2); 52 [50–55]	48.1 (1.8); 48 [47–50]	56.6 (1.5); 56 [55–58]	57.2 (1.8); 57 [56–59]	55.5 (1.9); 55 [54–57]
**IDM quintile**					
** 1 (very wealthy)**	4 (23.5%)	17 (18.5%)	12 (16.2%)	6 (6.9%)	23 (21.5%)
** 2 (wealthy)**	2 (11.8%)	12 (13%)	10 (13.5%)	18 (20.7%)	16 (15.0%)
** 3 (medium)**	6 (35.3%)	22 (23.9%)	18 (24.3%)	27 (31.0%)	21 (19.6%)
** 4 (deprived)**	2 (11.8%)	13 (14.1%)	13 (17.6%)	19 (21.8%)	12 (11.2%)
** 5 (very deprived)**	3 (17.6%)	28 (30.4%)	21 (28.4%)	17 (19.5%)	35 (32.7%)
**Ind_007—Unpolluted coasts for pollution**	2.5 (0.05); 2.5 [2.4–2.55]	4.6 (0.07); 4.7 [4.5–4.7]	2.0 (0.06); 2.0 [1.9–2.1]	5.0 (0.1); 5.1 [5.0–5.2]	1.8 (0.1); 1.9 [1.8–2.0]
**Ind_009 –Efficiency of water distribution**	53.7 (1.2); 54 [51–55]	53.6 (2.1); 53 [51–56]	56.9 (1.9); 57 [55–59]	(1.6), [55 [52–57]	53.1 (1.4); 53 [51–52]
**Ind_012—Unemployment rate**	15 (2.8); 15.5 [12.5–17.7]	16.8 (2); 16.7 [15.9–18.7]	11 (1.9); 10 [9.9–11.7]	17 (2); 17.4 [15.1–19.7]	18 (2.9); 18.6 [15.7–21.2]
**Ind_013—Employment rate**	51.7 (1.35); 52 [50.4–52.7]	50.1 (2.0); 51.2 [47.5–51.7]	51.3 (1.6); 50.7 [50.5–52.8]	50.1 (1.2); 50.4 [49–51]	49.8 (1.5); 49.9 [47.6–52.1]
**Ind_014—Employment rate over 54 years**	42.6 (7.2); 41 [38.5–45.9]	39.7 (8.5); 42.0 [39.8–45.2]	39.5 (7.9); 40.8 [31.3–43.1]	38.0 (4.8); 38.4 [35.8–45.9]	33.0 (7.5); 29.9 [27.4–37.7]
**Ind_015 –Young unemployment rate**	35.4 (6.8); 34.4 [29.4–41]	53.5 (4.9); 52.7 [49.6–57.5]	55.2 (14); 55.6 [42.5–68.1]	51.5 (11); 52 [44.9–59.6]	1.4 (8.5); 47.3 [42.8–53.5]
**Ind_018—Cultural demand**	21.8 (7.9); 18.2 [17.6–28.4]	9.3 (0.85); 9.3 [8.7–10.3]	3.8 (2.4); 2.9 [2.7–3.4]	24.6 (11.5); 23.7 [10.8–36.1]	7.2 (3.1); 7.5 [6.8–8.5]
**Ind_024—Degree of promotion of the cultural offer of state institutions**	308 (420); 198 [67–236]	45 (23); 38 [31–41]	73 (75); 40 [35–70]	838 (66); 941 [194–1232]	47 (19); 45 [36–48]
**Ind_027—Degree of diffusion of theatrical and musical entertainment**	58.0 (11.6); 52.1 [51.3–67.3]	45.2 (5.3); 44.4 [42.3–49.4]	23.2 (5.0); 22.9 [20.3–25.3]	25.3 (2.9); 26.5 [23.9–26.7]	39.6 (15.6); 38.6 [26.4–52.3]
**Ind_044—Air traffic index**	1222 (116); 1228 [1085–1615]	641 (23); 646 [621–661]	465 (25); 461 [459–488]	7.2 (10); 0 [0–19]	602 (110); 602 [589–618]
**Ind_052—Separate municipal waste**	49.5 (2.4); 49.5 [46.6–52.1]	43.6 (5.6); 44 [37.7–49.4]	54.2 (5.9); 56.1 [49.9–60]	62.5 (2.3); 63 [60.9–64.9]	59.5 (6.3); 60.5 [59.1–62.4]
**Ind_057—Difference between male and female employment rate**	17.2 (1.9); 16.8 [15.1–18.3]	14.9 (2.5); 15.2 [11.9–17.1]	14.8 (3.9); 16.1 [12.3–18.5]	17.5 (2.3); 18.3 [16.2–19.1]	23.7 (6.6), 22.8 [18.8–27.6]
**Ind_060 –Interruption of the electricity service**	3.6 (0.63); 3.5 [3.2–3.8]	2.3 (0.29); 2.3 [2–2.5]	2.5 (0.47); 2.5 [2.3–2.8]	4.02 (1.4); 3.8 [3.1–4.1]	3.5 (1.2); 3.3 [2.5–4.1]
**Ind_080—Energy produced from renewable sources**	2.8 (0.92); 2.7 [2.5–3.2]	7.2 (2.1); 6.7 [5.7–7.8]	18.9 (10.2); 13.7 [10.9–29.1]	8.0 (3.1); 6.8 [6.0–8.2]	5.9 (3.9); 5.8 [4.7–5.3]
**Ind_083—Municipal waste**	466 (26.6); 458 [438–492]	452 (28.4); 381 [368–394]	356 (28.9); 348 [329–379]	381 (12.3); 381 [369–393]	408 (22.1); 417.8 [389–420]
**Ind_105—Tourism rate**	4.9 (0.43); 4.8 [4.6–5.3]	5 (0.48); 4.9 [4.7–5.2]	6.4 (0.93); 6.6 [5.9–7.2]	2.8 (0.33); 2.6 [2.5–3.0]	3.5 (0.55); 3.8 [2.1–4.5]
**Ind_108—Participation of the population in the labour market**	61.1 (1.6); 60.3 [60–62.5]	60.4 (1.5); 59.6 [59.4–61.7]	57.8 (1.5); 57.7 [56.3–58.2]	60.5 (1.9); 61.2 [58.6–62.3]	55.7 (2.5); 56.1 [54.4–57.2]
**Ind_120—Weight of cooperative society**	6.9 (0.58); 6.8 [6.4–7.7]	2.5 (0.25); 2.5 [2.4–2.8]	3.3 (0.32); 3.2 [3.07–3.7]	9.2 (0.78); 8.9 [8.7–9.5]	2.1 (0.9), 2.1 [1.3–2.9]
**Ind_135—Micro criminality index**	5.3 (0.32); 5.2 [5.1–5.6]	4.1 (0.33); 4.0 [3.9–4.5]	3.0 (0.52); 2.9 [2.4–3.6]	5.0 (0.76); 4.9 [4.5–5.3]	4.7 (0.45); 4.7 [4.4–4.8]
**Ind_141—Hospital emigration**	4 (0.24); 4 [3.8–4.3]	4.8 (0.17); 4.9 [4.7–5]	5.7 (0.56); 5.5 [5.2–6.3]	4.9 (0.42); 5.1 [4.4–5.3]	3.7 (1.1); 3.5 [2.9–4.3]
**Ind_142—Childhood services**	50 (5); 50.7 [45.1–51]	31.4 (3.9); 28.8 [27.8–36.4]	35.7 (3.8); 38.5 [32.7–38.5]	22 (2.1); 22.7 [20.5–23]	37.8 (3.8); 39.5 [35.4–40]
**Ind_162—Funding risk**	3.5 (1.1); 3.1 [2.4–4.6]	4.3 (2.2); 3.1 [2.7–6.6]	4.3 (1.3); 4.8 [2.8–5.4[	2.9 (0.96); 2.5 [2.4–2.8]	3.7 (2.8); 2.8 [1.7–4.9]
**Ind_165—Tourism in not-summer period**	0.98 (0.13); 0.94 [0.91–1.13]	1.03 (0.09); 1.01 [0.97–1.11]	0.92 (0.25); 0.87 [0.75–1.14]	0.71 (0.076); 0.71 [0.65–0.75]	0.75 (0.15); 0.76 [0.65–0.78]
**Ind_168—Export index**	4.8 (0.85); 5 [4.4–5.2]	30 (7.5), 26 [23–36]	37 (24); 45 [12–59]	6.8 (4.1); 6.4 [5.7–7.8]	5.7 (3.5); 5.5 [4.8–7.2]
**Ind_175—Unemployment rate (male)**	14.5 (3.64); 13.7 [11.7–13.6]	16.8 (3.02); 16.5 [15.4–13.5]	10.8 (2.13); 10.6 [9.3–13.2]	15.2 (3.44); 14.7 [13.7–13.9]	16.8 (7.42); 13.8 [11.5–14.0]
**Ind_176—Unemployment rate (female)**	14.6 (1.90); 13.9 [13.1–15.3]	18.2 (2.79); 16.9 [16.6–20.7]	12.3 (2.49); 12.2 [10.8–14.1]	18.7 (2.9); 19.7 [16.7–20.8]	22.0 (2.41); 20.1 [19.3–20.6]
**Ind_177—Employment rate (male)**	61.3 (2.59): 60.7 [59.7–62.9]	57.0 (2.64); 57.3 [55.3–59.9]	59.1 (3.12); 59.7 [57.3–61.9]	59.3 (2.15); 58.6 [57.8–60.2]	56.7 (5.59); 58.2 [54.0–61.1]
**Ind_178—Employment rate (female)**	43.4 (1.28); 43.9 [42.9–44.4]	41.9 (2.85); 42.7 [39.9–43.3]	43.5 (1.92); 43.8 [43.2–45.2]	41.4 (2.24); 41.6 [39.9–42.4]	33.0 (2.06); 33.7 [31.7–34.2]
**Ind_232—Percentage of municipal waste disposed of in landfills**	45 (16); 33.5 [33–64.2]	90 (10.6); 95.8 [77.5–99.1]	60.5 (13); 69 [44.8–70]	214 (14.3); 224 [197.4–225]	29.7 (13.7); 26 [16.3–45.6]
**Ind_239—Forests surface**	3731 (2884); 2795 [1891–7806]	4087 (3160); 3062 [2071–8551]	4757 (3678); 3564 [2411–9952]	1076 (832); 806 [545–2251]	2081 (799); 1976 [799–2751]
**Ind_241—Enrolment gross rate in the business register**	6.4 (0.25); 6.4 [6.1–6.6]	6.3 (0.38); 6.3 [6.2–6.4]	5.9 (0.46); 6 [5.3–6.2]	6.15 (0.36); 6.2 [5.9–6.4]	5.9 (1.77); 5.9 [5.8–6]
**Ind_242—Enrolment rate in the business register**	0.52 (0.31); 0.54 [0.31–0.63]	0.82 (0.34); 0.96 [0.44–1.1]	1.3 (2.03); 0.51 [0.20–1.2]	0.88 (0.34); 0.95 [0.49–1.2]	0.79 (0.33); 0.81 [0.52–1.1]
**Ind_255—Forests surface burned by fire**	0.5 (0.48); 0.3 [0.21–0.65]	0.4 (0.36); 0.2 [0.13–0.53]	0.8 (0.69); 0.5 [0.29–0.90]	0.7 (0.63); 0.4 [0.31–0.92]	0.9 (0.12); 0.7 [0.45–0.98]
**Ind_265—Air quality monitoring**	3.4 (0.2); 3.4 [3.2–2.5]	3.2 (0.23); 3.3 [3–3.4]	3.5 (0.5); 3.2 [3.1–3.8]	1.9 (0.22); 1.8 [1.7–1.9]	3.3 (0.25); 3.2 [2.9–3.4]
**Ind_278—Flood risk population**	6.1 ab / km^2^	1.43 ab / km^2^	0.89 ab / km^2^	5.2 ab / km^2^	5.6 ab / km^2^
**Ind_279—Rate of reported thefts**	18.8 (1.7); 19.2 [17.5–24.7]	21.4 (2.3); 21.7 [20.8–24.9]	15.8 (2.1); 16.2 [14.6–24.7]	8.5 (1.17); 8.3 [7.8–24.7]	17.2 (1.9); 16.8 [15.4–25.4]
**Ind_280—Rate of reported robberies**	0.37 (0.05); 0.37 [0.32–0.42]	0.33 (0.04); 0.33 [0.29–0.37]	0.38 (0.12); 0.38 [0.37–0.46]	0.11 (0.02); 0.11 [0.08–0.12]	0.29 (0.07); 0.29 [0.25–0.47]
**Ind_281—Homicides rate**	0.9 (0.55); 0.89 [0.36–1.43]	1.2 (0.77); 0.91 [0.6–2.1]	5.1 (1.8); 5.6 [3.8–5.7]	1.04 (0.57); 1.21 [0.61–1.22]	1.07 (0.81); 0.73 [0.64–1.09]
**Ind_414—Taking charge of all users of childcare services**	13.6 (2); 13.1 [12.9–13.6]	16 (3.4); 14.5 [14–18.3]	15.2 (2.3); 14.3 [13.9–14.6]	11.6 (1.3); 10.8 [10–13.4]	12.6 (1.5); 11.8 [10.2–14.2]
**Ind_415—Elderly in social assistance**	1.9 (0.08); 1.9 [1.7–1.9]	2.25 (0.11); 2.2 [2.1–2.3]	2.9 (0.21); 2.8 [2.7–2.9]	3.68 (0.14); 3.7 [3.6–3.8]	3.89 (0.18); 4.1 [3.5–4.3]
**Ind_445—Index of accessibility to urban nodes**	77.5 (18); 72 [66.7–81.7]	65.4 (11.2); 67 [64.8–69.2]	58.1 (12); 62 [55–59]	70.6 (14); 65 [61 – 72]	120.4 (21); 116 [110–123]

### NBRM for African swine fever in wild boar

As shown in S3 Table, 22 predictors for ASF in WB between 2015and 2018 were identified and following elimination of 14 covariates for multicollinearity, the results obtained by the mixed-effect NBRM are shown in Table 3 and expressed as the (OR_adj_, [95% CI]) and p-value. Growing cultural demand (Ind_018) and the energy produced by renewable sources (Ind_080) were valid indicators for a corresponding low probability of ASF in WB, with OR_adj_ = 0.98 ([95% CI = 0.95–0.99], p = 0.022), OR_adj_ = 0.57 ([95% CI = 0.35–0.92], p = 0.023), respectively. With regards to those variables which were risk indicators, an increase of one point in the micro-criminality index (Ind_135) increased the risk of ASF in WB by 93% (OR_adj_ = 1.93, [95% CI = 1.89–1.97], p = 0.001), with a similar effect noted with the flood risk population (Ind_278) (OR_adj_ = 1.79, [95% CI = 1.65–1.97], p = 0.027), and the amount of differentiated waste (Ind_052) (OR_adj_ = 1.49, [95% CI = 1.11–1.99], p = 0.009). Furthermore, a statistically significant effect in predicting the number of disease cases was noted found for the forest surface (Ind_239), with an OR_adj_ of 1.17 ([95% CI = 1.08–1.20], p < 0.0001). An increase of one point in the rate of reported thefts (Ind_279) doubled the probability of observing one ASF WB case in the same municipality (OR_adj_ = 2.65, [95% CI = 1.90–3.69], p < 0.0001). In municipalities with a high rate of employment (Ind_013), the probability of ASF cases in WB decreased by 20% for each point increment in the rate (OR_adj_ = 0.81, [95% CI = 0.99–1.0.66], p = 0.041). The Likelihood-ratio test (LR chi^2^ = 369.78, prob > chi^2^ = 0.0001), favoured the mixed-effect NBRM against the mixed-effect Poisson regression model. Data used for external validation (between 2011 and 2014) are reported in Table 1. The predicted performance of the final model for ASF in WB was tested and shown to be able to predict the outcome correctly, according to internal validation criteria (residual mean = 3.99*10^−6^, SD = 1.36*10^−6^, Spearman’s correlation coefficient = 0.834, p < 0.0001) and external validation (residual mean = 5.16*10^−3^, SD = 3.25*10^−3^, Spearman’s correlation coefficient = 0.893, p < 0.0001). The root mean square tests were insignificant for both datasets (Root MSE = 0.232, p = 0.53; Root MSE = 0.198, p = 0.64, respectively), indicating no evidence of failure. Fig 1C and 1D are a graphical representation of the model’s residuals and agreement between the observed values within the ‘test dataset’.

### NBRM for contagious agalactia

A total of 21 evaluable predictors for the risk of CA were identified (S3 Table), with 10 covariates being deleted from the final NBRM (Table 3) and statistically significant effects being detected. Increasing the number of farms by 10, increases the number of CA cases by 18% (OR_adj_ = 1.18, [95% CI = 1.10–1.26], p < 0.0001), while an increase in the number of animals (for every further 100 animals) was associated with a 0.4% increase in the number of cases (OR_adj_ = 1.01, [95% CI = 1.01–1.03], p = 0.038). The number of cases grew by 16% for every 5-year increase in the age of the farmer (OR_adj_ = 1.16, [95% CI = 1.01–1.33], p = 0.029). The risk was low when the farmer was female, with an OR_adj_ equal to 0.52 ([95% CI = 0.37–0.74], p < 0.0001). With regards to the ISTAT indicators, a protective role was played by cultural demand (Ind_018) (OR_adj_ = 0.50, [95% CI = 0.39–0.65], p < 0.0001), and the forest surface (Ind_239) (OR_adj_ = 0.98, [95% CI = 0.96–0.99], p = 0.001). The proportion of CA cases increased by 6% with the growing amount of municipal waste (Ind_083) (OR_adj_ = 1.06, [95% CI = 1.02–1.10], p = 0.027) and by 45% with the increase in flood risk population (Ind_278) (OR_adj_ = 1.22, [95% CI = 1.03–1.45], p = 0.023). Two indicators related to criminality were valid predictors for disease cases; the number of CA cases in the municipality increased by 28% with a one-point increment in the micro-criminality index (Ind_135) (OR_adj_ = 1.282, [95% CI = 1.03–1.60], p = 0.028) and by 3% in reported thefts (Ind_279) (OR_adj_ = 1.03, [95% CI = 1.01–1.05], p = 0.004). The likelihood-ratio test (LR chi^2^ = 427.66, prob > chi^2^ = 0.0001), favoured the mixed-effect NBRM against the mixed-effect Poisson regression model. The predicted performance of the final model for CA was tested and shown to be able to predict the outcome correctly, both for internal (residuals’ mean = 3.12*10^−5^, SD = 0.99*10^−5^, Spearman’s correlation coefficient = 0.845, p < 0.0001) and external validation (residuals’ mean = 6.18*10^−3^, SD = 4.11*10^−3^, Spearman’s correlation coefficient = 0.799, p < 0.0001). The root mean square tests were insignificant for both datasets (Root MSE = 0.412, p = 0.33; Root MSE = 0.399, p = 0.18, respectively), indicating no evidence of failure. Fig 1E and 1F are a graphical representation of the model’s residuals and agreement between observed values within the ‘test dataset’.

### NBRM for West nile disease

Risk factors that had a p-value ≤ 0.20 in the univariable analysis are presented in S3 Table. A total of 15 evaluable variables were included in the model for WND. The results of the final NBRM are reported in Table 3, including the 5 covariates which play a role in the WND risk identification. All variables not typically indicative of rural areas were associated with a lower risk of WND cases; each point increment in the employment rate (Ind_013) resulted in a reduction of 2% in WND cases noted (OR_adj_ = 0.98, [95% CI = 0.89–0.99], p < 0.0001), growing cultural demand (Ind_018) was associated with a 33% decrease in the number of cases (OR_adj_ = 0.67, [95% CI = 0.47–0.95], p = 0.025), and each point increment in the indicator for energy produced from renewable sources (Ind_080) was associated with a 69% reduction in the probability of a new WND case (OR_adj_ = 0.32, [95% CI = 0.21–0.48], p < 0.0001). The associated identified risk of WND grew with each 100 inhabitants exposed to flood risk (by km^2^) (OR_adj_ = 1.13, [95% CI = 1.02–1.25], p = 0.016). A lower identified risk of disease spread in forest area was confirmed by the fact that increasing the forest surface area decreases the number of WND cases (OR_adj_ = 0.51, [95% CI = 0.32–0.81], p = 0.005). The likelihood-ratio test (LR chi^2^ = 366.51, prob > chi^2^ = 0.0001), favoured the mixed-effect NBRM. The predicted performance of the final model for WND was tested and shown to be able to predict the outcome appropriately, according to validation criteria (internal validation: residual mean = 2.99*10^−7^, SD = 1.25*10^−7^, Spearman’s correlation coefficient = 0.696, p < 0.0001; external validation: residual mean = 1.09*10^−3^, SD = 0.84*10^−3^, Spearman’s correlation coefficient = 0.705, p < 0.0001). The root mean square tests were insignificant for both datasets (Root MSE = 0.774, p = 0.91; Root MSE = 0.612, p = 0.55, respectively), indicating no evidence of failure. Fig 1G and 1H are a graphical representation of the model’s residuals and agreement between the observed values within the ‘test dataset’.

### NBRM for bluetongue

Of the 20 possible covariates selected by p-value ≤ 0.20 in the univariable analysis (S3 Table), only the 8 variables that were able to properly predict the number of BT outbreaks were included in the final NBRM; the results are reported in Table 3. Growing numbers of susceptible animals in each municipality increased the probability of identifying an area as being at risk for BT outbreaks (OR_adj_ = 1.16, [95% CI = 1.03–1.31], p = 0.013). Comparing the rural areas with more developed regions, seemed to confirm the higher probability of identifying the former as being at risk of BT outbreaks; high levels of cultural demand (Ind_018) (OR_adj_ = 0.98, [95% CI = 0.97–0.98], p < 0.0001), the tourism rate (Ind_105) (OR_adj_ = 0.95, [95% CI = 0.91–0.97], p = 0.002), and air quality monitoring (Ind_265) (OR_adj_ = 0.63, [95% CI = 0.45–0.77], p < 0.0001) corresponded to a decrease in the number of BT outbreaks. In those municipalities with growing unemployment rates (Ind_012), the probability of observing an additional BT outbreak increased by 21% (OR_adj_ = 1.21, [95% CI = 1.02–1.44], p = 0.029), and by 31% with an increase in the rate of homicides (Ind_281) (OR_adj_ = 1.31, [95% CI = 1.06–1.63], p = 0.001). A higher proportion of population at flood risk was associated with a 27% increase in the probability of an outbreak (OR_adj_ = 1.27, [95% CI = 1.04–1.56], p = 0.019). As previously demonstrated by several studies, increasing the forest area was associated with decreasing the number of BT outbreaks (OR_adj_ = 0.45, [95% CI = 0.22–0.76], p = 0.011), as forests are not favourable habitats for this disease [36] [77] [78] [79] [80]. The Likelihood-ratio test (LR chi^2^ = 425.98, prob > chi^2^ = 0.0001), favoured the mixed-effect NBRM against the mixed-effect Poisson regression model. The residual’s means obtained from ‘training dataset’ and the ‘test dataset’ suggest that the final model could be able to predict the outcome properly (residual mean = 3.65*10^−7^, SD = 2.10*10^−7^; residual mean = 3.98*10^−2^, SD = 1.76*10^−2^, respectively). The predicted CA number of cases are highly correlated with the observed ones, both in internal (Spearman’s correlation coefficient = 0.920, p < 0.0001) and external validation (Spearman’s correlation coefficient = 0.853, p < 0.0001).The root mean square tests were insignificant for both datasets (Root MSE = 0.732, p = 0.12; Root MSE = 0.589, p = 0.37, respectively), indicating no evidence of failure. Fig 1I and 1J are a graphical representation of the model’s residuals and agreement between observed values within the ‘test dataset’.

### NBRM for trichinellosis

Risk factors with p-value ≤ 0.20 in the univariable analysis are presented in S3 Table. An overall 28 variables related to trichinellosis were considered for inclusion in the final mixed-effects NBRM, of which 12 covariates were included in the final model, after multicollinearity detection and model validation. As shown in Table 3, increasing the number of farms by 10 in each municipality seemed to be associated with a 0.9% increase in the number of disease cases (OR_adj_ = 1.01, [95% CI = 1.01–1.02], p < 0.0001). At the same time, the number of animals contributed to a 7% increase in cases per every additional 100 animals in the same municipality (OR_adj_ = 1.07, [95% CI = 1.02–1.12], p = 0.006). For every 5-year increase in age, older farmers had an associated increased probability of trichinellosis cases by 19%, compared to younger farmers (OR_adj_ = 1.19, [95% CI = 1.05–1.35], p = 0.008). Female farmers were associated with a lower probability than males farmers (OR_adj_ = 0.89, [95% CI = 0.80–0.98], p < 0.0001). Comparing the first DMI level (lower deprivation) with others, the OR_adj_ suggests an increased probability of ASF spread in farms, with statistically significant results between DMI level-1 and DMI level-4 (OR = 2.70, [95% CI = 1.45–6.09], p = 0.003), or DMI level-5 (OR = 1.64, [95% CI = 1.22–2.19], p = 0.001). An increment in the rate of employment (Ind_013), cultural demand (Ind_018), and renewable energy produced (Ind_080) resulted in a decrease in the number of trichinellosis cases, in the same municipality by 8% (OR_adj_ = 0.92, [95% CI = 0.87–0.97], p = 0.004), 18% (OR_adj_ = 0.82, [95% CI = 0.72–0.94], p = 0.003), and 87% (OR_adj_ = 0.13, [95% CI = 0.04–0.42], p = 0.001), respectively. Increasing levels of micro-criminality (Ind_135) and reported thefts (Ind_279) increased the number of cases by 16% (OR_adj_ = 1.16, [95% CI = 1.03–1.31], p = 0.013) and 30% (OR_adj_ = 1. 30, [95% CI = 1.11–1.51], p = 0.001) for each index point increment, respectively. Increased tourism in the non-summer period (Ind_165), expressed as the number of days of tourist presence (Italians and foreigners), generated a 65% higher probability of observing cases (OR_adj_ = 1.65, [95% CI = 1.23–2.18], p = 0.001), in the same municipality. The number of trichinellosis outbreaks grew two times with each point increment in the homicide rate (Ind_281), as indicated by OR_adj_ of 2.31 ([95% CI = 1.11–4.80], p = 0.025). The Likelihood-ratio test (LR chi^2^ = 245.382, prob > chi^2^ = 0.0001), favoured the mixed-effect NBRM. The predicted performance of the final model for trichinellosis was tested based on ‘training dataset’ and ‘test dataset’ and shown to be able to predict the outcome correctly, according to validation criteria (residual mean = 5.21*10^−7^, SD = 3.27*10^−7^, Spearman’s correlation coefficient = 0.852, p < 0.0001; residual mean = 10.36*10^−3^, SD = 6.88*10^−3^, Spearman’s correlation coefficient = 0.814, p = 0.001; respectively). The root mean square tests were insignificant for both datasets (Root MSE = 0.585, p-value = 0.36; Root MSE = 0.692, p-value = 0.24, respectively), indicating no evidence of failure. Fig 1K and 1L are a graphical representation of model’s residuals and agreement between the observed values within the ‘test dataset’.

### NBRM for cystic echinococcosis

A total of 22 explanatory variables have been considered for inclusion in the model, aimed to identify the areas at high risk for CE (S3 Table). Using the inclusion criteria in the NBRM, a total of 15 explicatory covariates were detected and their role in risk identification is shown in Table 3. The number of animals and farms in each municipality were significant risk indicators and showed a tendency to positively describe the outbreak with an OR_adj_ of 1.01 at p < 0.0001 ([95% CI = 1.01–1.09]) calculated per 100 animals, and an OR_adj_ of 1.009 at p = 0.036 ([95% CI = 1.01–1.18]), per 10 farms. An increased age of the farmer appeared to have a protective effect against event probability (OR_adj_ = 0.98; [95% CI 0.96–0.99], p = 0.001), with a similar effect noted for the female sex (OR_adj_ = 0.84; [95% CI = 0.82–0.86], p < 0.0001). Comparing the first MDI level (lower deprivation) with others, the OR_adj_ suggested an increasing probability of CE, with statistically significant results, comparing the MDI level 1 and the MDI level 4 (OR_adj_ = 1.95, IC 95% [1.02–3.81], 0.047), or MDI level 5 (OR_adj_ = 3.73, IC 95% [3.54–3.94], p < 0.0001). With regards to the ISTAT indicators for sociology, the NBRM results highlighted a decreasing risk identification in farms located in municipalities with growing employment rate (Ind_013), cultural demand (Ind_018), air traffic index (Ind_044), tourism rates (Ind_105) and hospital emigration (Ind_141), described by an OR_adj_ of 0.91 ([95% CI = 0.86–0.97], p = 0.003), 0.84 ([95% CI = 0.67–0.86], p = 0.004), 0.98 ([95% CI = 0.97–0.99], p < 0.0001), 0.94 ([95% CI = 0.92–0.97], p < 0.0001) and 0.72 ([95% CI = 0.63–0.83], p < 0.0001), respectively. All other indicators such as micro-criminality index (Ind_135), number of inhabitants in the population at flood risk (Ind_278), rates of reported theft (Ind_279) and robberies (Ind_280) and homicide rate (Ind_281) had an OR_adj_ > 1, indicating a role in favouring an increase in CE cases. The Likelihood-ratio test (LR chi^2^ = 398.41, prob > chi^2^ = 0.0001), favoured the Mixed-effect NBRM. The predicted performance of the final model for CE was tested and shown to be able to predict the outcome accurately, according to validation criteria The mean of the residual’s distribution is 3.77*10^−7^ with a SD of 2.09*10^−7^. The predicted values were highly correlated with the observed ones (Spearman’s correlation coefficient = 0.903, p-value = 0.001). The external validation performed on “test dataset” suggested that the model adequately fits the observed values and their correlation is high as in the internal validation (Spearman’s correlation coefficient = 0.872, p-value = 0.001). The residuals had an average of 2.65*10^−2^ with an SD of 2.74*10^−3^. The root mean square tests were insignificant for both datasets (Root MSE = 0.355, p-value = 0.48; Root MSE = 0.430, p-value = 0.18, respectively), indicating no evidence of failure. Fig 1M and 1N are a graphical representation of the model’s residuals and agreement between the observed values within the “test dataset”.

## Discussion

This study described six different animal disease trends, their current situations in Sardinia, and attempted to relate them with social and demographic statuses. In this regard, in 2014, the OIE Guidelines affirmed that a disease control programme should take into account socio-economic status and consider different non-financial factors (i.e. social, cultural, and religious) affecting the livelihood and well-being of animal owners such as pastoralists, farmer communities, or small-scale backyard producers. These factors can be important incentives for participation or non-compliance and ultimately impact the success of the programme. Farms located in areas with socio-economic problems are particularly vulnerable to disease because of the expense and the absence or unsuitability of animal health and production inputs [39]. The necessity to include social and economic factors in risk analysis is particularly felt in Sardinia, where breeding has always been one of the major economic resources, with sheep breeding, at present, representing the largest sheep heritage in Italy. As such, the agro-pastoral tradition is rooted in the Sardinian municipalities and is part of their culture, being influenced also by other local mores and the various social factors that distinguish them. At the same time, the great concentration of animals favours the development and spread of various diseases; the condition of the island (i.e. isolation, climate, land type) also favours the endemicity of some of these, as well as epidemic seasons. However, studies based on social factors involved in disease spread are very rare and this may be a valid set of factors to consider. Of the 44 ISTAT Territorial Indicators used for the purpose of this work, 22 of these described the diseases spread in Sardinia. Comparing the six diseases and evaluating the contemporary presence of one or more covariates in the final models, it is possible to observe that the most common indicator was cultural demand (Ind_018), followed by the employment rate (Ind_013), and the proportion of inhabitants at flood risk (Ind_278). Some indicators such as MDI-level, numbers of farms and animals, the age and sex of the farmer, the micro-criminality index (Ind_135), and rate of reported thefts (Ind_279) were common in models which tried to describe the spread of ASF, CA, trichinellosis, and CE. Some others such as the forest surface (Ind_239) and the energy produced from renewable sources (Ind_080) were common in BT, WND, and ASF in WB. Tourism in non-summer seasons (Ind_165) was a valid predictor for ASF and trichinellosis, while hospital emigration only had a statistically significant role in CE risk identification. The demographic characteristics analysed with other risk indicators highlighted that male farmers were more frequently associated with a higher level of disease risk. Breeding managed by young farmers seemed to be a valid indicator in defining high-risk areas for ASF and CE, whereas the older farmers were more susceptible to identify areas at high risk for trichinellosis and CA. This trend may be related to the timing of the last public health programme was executed in Sardinia: the first CE and ASF cases date back 40 years, so the resulting information campaigns would have also reached the oldest breeders. However, it’s important to take into account the possible changes in breeding practices during the last 40 years. Given that a relationship between health status and class, poverty, or material deprivation has been demonstrated, the MDI was used to evaluate the socio-economic status and the relationship between habits, customs, and disease development, assuming a relationship between these conditions [81]. In three of the disease models, the higher MDI-level value was strongly associated with high risk identification. High municipality cultural demand described a condition where the risk of disease was low, whereas a low employment rate was an indicator of a high level of disease risk. Generally, cultural demand was proportional to the urbanisation level and the latter was directly proportional to the number of employed people [40]. These two factors in association with the proportion of inhabitants in the flood risk population, could describe rural areas where the risk of disease is higher. Obviously, these factors are not directly associated with disease development, but they may able to describe a condition where the disease could spread [82]. Given the results, it is remarkable that the diseases with the most social factors in common are ASF and trichinellosis. The social factors involved in these two diseases and the similarity between them was studied by Pozio et al. in 2006, where the authors affirmed that probably the occurrence of trichinellosis cases was likely strictly correlated to illegal free-ranging pig breeding [22]. The fundamental role of free-ranging pigs in ASF transmission and persistence has previously been demonstrated [6] [7] [31] [32] [83] [84] [85]. This would suggest that in Sardinia the two diseases are linked both from an epidemiological and a socio-economic point of view. Although the present study was conducted based on data from Sardinia, and despite the use of specific social variables for Italy, and also noting that the results of the models are epidemic events of the different diseases, the results obtained here may be a point of departure for wider reflection. Interpretation of disease risks should not be limited to the dissemination of recommended health protocols (e.g. ‘public health programme’), but should also inspire social research in other countries, where cultural and economic conditions are similar, and particularly more so in those with different conditions, in order to identify specific indicators. Most limitations of this study are related to its retrospective nature (i.e. data traceability, accuracy, and underreporting). The checks carried out before the analysis process due to the problems related to BDN registration may have limited, at least partly, the generation of possible selection bias. For all the diseases considered, specific Italian surveillance programmes were in place, aiming for the early detection of each case. As such, the probability of underreporting cases is exceedingly low, but precisely valuable. Moreover, some variables were used as proxies for factors which seem to be important in influencing and/or limiting farm management and biosecurity. Not all the possible socio-economic factors that might affect animal health were included in the final models and some final predictors might have played the role of surrogates for other variables not included. Using a proxy may provide a reflected measure, although with less accuracy, of features that cannot be directly measured. However this can influence the interpretation and the use of the results. For example, the significant role of hospital emigration (Ind_141) as indicator in disease occurrence should not be interpreted as direct effect, but as proxy for a low sanitary context, such as high unemployment rate (Ind_012) could give an idea of deprived and poor social context. Other limitations of the present study stem from its lack of understanding of the qualitative behaviour change of farmers. Recent studies have suggested that each farmer develops a different control strategy depending on their situation and disease understanding [2] [9]. For example, Elbers et al. in 2010 [86] suggested that the previous experience of having BT outbreaks in farms was associated with a higher probability of vaccinating their livestock, as confirmed in Sardinia by Cappai et al. eight years later [36]. Furthermore, as pointed out by Barnes et al. in 2015 [87], interaction between economic and socio-psychological factors could play a key role in farmer decision making, such as the influence from different actors (i.e. veterinarians, society, industry) on the farmer’s behaviour. Moreover, in Sardinia, traditional breeding practices are rooted in the agro-pastoral culture and dates back many decades [5] [88]. Therefore, it is likely that the influence of farming culture, tradition, and family farm thinking often drive farm practices and may change over time and between farmer generations. As expressed very well by Craddock and Hinchliffe in 2015 in their One Health concept, in some cases the real importance is not just to determine the disease cases, but perceptions of disease and how they fit within broader social imaginaries of health and disease [38]. Furthermore, the effects of disease on livestock include direct effects on productivity, disease control costs, and constraints on livestock management, including limitations on species and breed choices, and human welfare effects [89]. The need to better understand the role of socio-economic status, demographic factors, and farm culture in influencing the farmer’s behaviour and the spread of disease cannot be negletted in a One Health perspective [2]. As noted by Barnes et al. in 2015 [87], there is also a critical knowledge gap in the interactions between economic and socio-psychological factors on farmer decision making. The novel approach to analyse the connection between animal diseases and socio-economic factors proposed in the present work suggests that for spreading the disease there could be also responsible human factors, giving a new tools to fight with them and predict the diseases direction. The importance of the results lies in making researchers, veterinarians, authorities and all involved stakeholders aware of what, why, and how diseases are relevant or not in particular regional contexts. Furthermore, the present work emphasize the importance of real and direct contact with local realities, not just for disease understanding but that of transmission and risk behaviours.

## Conclusion

The present work highlights the role of a few socio-economic factors as indicators to identify macro-areas or municipalities at the greatest risk of farm animal-related diseases. This important information should be considered in for disease control planning, in order to put into place targeted and specific programmes for each territory, to identify the major risk areas by considering the features typical for each disease, and the human population conditions which would present an increased risk. The goal for greater interdisciplinary teamwork has also been pursued by different studies in the field of animal disease. However, institutional boundaries and disciplinary norms can make attractive but distant prospects. The integrated vision that human beings, animals, and the environment are linked plays a key role in responding to the current challenges facing the world. Understanding these links is necessary to better recommend strategies that predict, prevent, respond to, and mitigate the spread of diseases, taking into account an environmental and socioeconomic backgrounds that may not change in a short period of time. The goal of One Health based on Healthy publics programmes is evidently ‘in the making’ and as such is fragmentary, and conditioned by geographic and historic specificities and expertise, and thus requires the implementation of new and challenging work practices. The main Health public purpose should instigate a radical change, not only in public and general thought, or educational programmes, but also in their engagement in research. To achieve these goals, a collaborative environment between scientists from veterinary epidemiology, animal welfare and social science backgrounds is essential in order to better understand behaviours and the decision making process of not only farmers, but also humans in general. Additionally, more epidemiological theoretical studies, that incorporate dynamic and detailed human behaviour and social status into the modelling of infectious diseases continue to be necessary to identify risk factors that require further investigation. This work could be a valid instrument to guide the future in deep researches and, subsequently, the decision maker.

## Supporting information

S1 Table List of variable collected by the macro-area of arguments. (DOCX)

S2 Table Italian cases distribution (excluding Sardinia), by study-year (2011–2018) and type of disease. (DOCX)

S3 Table Final decision of inclusion in the final Negative Binomial Regression Models (NBRMs) for each variables collected. Data are divided by disease (corresponding to different NBRM) and presented as P-value resulting from univariable analysis, and final decision of inclusion (Yes) or exclusion (Not), based on p-value, variance inflation factor (i.e. multicollinearity evaluation), experimental requirements.
(DOCX)
