# Correction: Socio-economic factors as indicators for various animal diseases in Sardinia

**DOI:** 10.1371/journal.pone.0220945

**Published:** 2019-08-05

**Authors:** Federica Loi, Alberto Laddomada, Annamaria Coccollone, Elena Marrocu, Toni Piseddu, Giovanna Masala, Ennio Bandino, Stefano Cappai, Sandro Rolesu

There is an error in Table 3. Table 3 is a duplicate of Table 2. Please see the complete, correct Table 3 here.

**Table 3:** Negative Binomial Regression model results for African Swine Fever in domestic pigs, African wine fever in wild boar, Contagious Agalactia, West Nile disease, Bloutongue, Trichinellosis and Cistic Echinococcosis as outcome. Data are reported as Adjusted Odds Ratio (OR_adj_), Confidence Interval at 95% and p-value.

OUTCOME: Cases of African Swine Fever in domestic pigs	OR_adj_	95% CI	p-value
N. farms (by 10 farms)	1.08	[1.02–1.11]	0.005
N. animals (by 100 animals)	1.01	[1.01–1.02]	< 0.0001
Age of the farmer (by 5 years old)	0.80	[0.70–0.84]	< 0.0001
Sex of the farmer (female vs male)	0.51	[0.33–0.82]	0.005
IDM quintile 1 (very wealthy) 2 (wealthy) 3 (medium) 4 (deprived) 5 (very deprived)	Rif. 0.89 2.04 2.23 2.64	[0.56–1.37] [1.70–2.44] [1.70–2.95] [1.90–3.69]	0.564 < 0.0001 < 0.0001 < 0.0001
Ind_013—Employment rate	0.77	[0.62–0.96]	0.019
Ind_018—Cultural demand	0.95	[0.93–0.98]	0.001
Ind_135—Micro criminality Index	1.46	[1.07–1.98]	0.016
Ind_165—Tourism in not-summer period	1.47	[1.31–1.65]	< 0.0001
Ind_278—Flood risk population (by 100 inhabitants/km^2^)	1.48	[1.11–1.99]	0.009
Ind_279—Rate of reported thefts	1.58	[1.51–1.28]	< 0.0001
Ind_280—Rate of reported robberies	1.07	[1.03–1.13]	0.001
**OUTCOME:** **Cases of African Swine Fever in wild boar**	**OR**_**adj**_	**95% CI**	**p-value**
Ind_013—Employment rate	0.81	[0.66–0.99]	0.041
Ind_018—Cultural demand	0.98	[0.95–0.99]	0.022
Ind_052—Municipal differentiated waste	1.49	[1.11–1.99]	0.009
Ind_080—Energy produced from renewable sources	0.57	[0.35–0.92]	0.023
Ind_135—Micro criminality Index	1.93	[1.89–1.97]	0.001
Ind_239—Forests surface (100 hectars)	1.17	[1.08–1. 20]	< 0.0001
Ind_278—Flood risk population (by 100 inhabitants/km^2^)	1.79	[1.65–1.97]	0.027
Ind_279—Rate of reported thefts	2.65	[1.90–3.69]	< 0.0001
**OUTCOME:** **Cases of Contagious Agalactia**	**OR**_**adj**_	**95% CI**	**p-value**
N. farms (by 10 farms)	1.18	[1.10–1.26]	< 0.0001
N. animals (by 100 animals)	1.01	[1.01–1.03]	0.038
Age of the farmer (by 5 years old)	1.16	[1.01–1.33]	0.029
Sex of the farmer (female vs male)	0.52	[0.37–0.74]	< 0.0001
Ind_018—Cultural demand	0.50	[0.39–0.65]	< 0.0001
Ind_083—Municipal waste	1.06	[1.02–1.10]	0.027
Ind_135—Micro criminality Index	1.28	[1.03–1.60]	0.028
Ind_165—Tourism in not-summer period	1.58	[1.41–1.82]	0.002
Ind_239—Forests surface (100 hectars)	0.98	[0.96–0.99]	0.001
Ind_278—Flood risk population (by 100 inhabitants/km^2^)	1.22	[1.03–1.45]	0.023
Ind_279—Rate of reported thefts	1.03	[1.01–1.05]	0.004
**OUTCOME:** **Cases of West Nile disease**	**OR**_**adj**_	**95% CI**	**p-value**
Ind_013—Employment rate	0.98	[0.89–0.99]	< 0.0001
Ind_018—Cultural demand	0.67	[0.47–0.95]	0.025
Ind_080—Energy produced from renewable sources	0.32	[0.21–0.48]	< 0.0001
Ind_239—Forests surface (100 hectars)	0.51	[0.32–0.81]	0.005
Ind_278—Flood risk population (by 100 inhabitants/km^2^)	1.13	[1.02–1.25]	0.016
**OUTCOME:** **Cases of Bluetongue**	**OR**_**adj**_	**95% CI**	**p-value**
N. animals (by 100 animals)	1.16	[1.03–1.31]	0.013
Ind_012—Unemployment rate	1.21	[1.02–1.44]	0.029
Ind_018—Cultural demand	0.98	[0.97–0.98]	< 0.0001
Ind_105—Tourism rate	0.95	[0.91–0.97]	0.002
Ind_239—Forests surface (100 hectars)	0.45	[0.22–0.76]	0.011
Ind_265—Air quality monitoring	0.63	[0.45–0.77]	< 0.0001
Ind_278—Flood risk population (by 100 inhabitants/km^2^)	1.27	[1.04–1.56]	0.019
Ind_281—Homicide rate	1.31	[1.06–1.63]	0.001
**OUTCOME:** **Cases of Trichinellosis**	**OR**_**adj**_	**95% CI**	**p-value**
N. farms (by 10 farms)	1.01	[1.01–1.02]	< 0.0001
N. animals (by 100 animals)	1.07	[1.02–1.12]	0.006
Age of the farmer (by 5 years old)	1.19	[1.05–1.35]	0.008
Sex of the farmer (female vs male)	0.89	[0.80–0.98]	< 0.0001
IDM quintile 1 (very wealthy) 2 (wealthy) 3 (medium) 4 (deprived) 5 (very deprived)	Rif. 0.84 1.77 2.70 1.64	[0.32–2.28] [1.25–2.51] [1.45–6.09] [1.22–2.19]	0.721 0.001 0.003 0.001
Ind_013—Employment rate	0.92	[0.87–0.97]	0.004
Ind_018—Cultural demand	0.82	[0.72–0.94]	0.003
Ind_080—Energy produced from renewable sources	0.13	[0.04–0.42]	0.001
Ind_135—Micro criminality Index	1.16	[1.03–1.31]	0.013
Ind_165—Tourism in not-summer period	1.65	[1.23–2.18]	0.001
Ind_279—Rate of reported thefts	1.30	[1.11–1.51]	0.001
Ind_281—Homicides rate	2.31	[1.11–4.80]	0.025
**OUTCOME:** **Cases of Cistic Echinococcosis**	**OR**_**adj**_	**95% CI**	**p-value**
N. farms (by 10 farms)	1.01	[1.01–1.18]	0.036
N. animals (by 100 animals)	1.01	[1.01–1.09]	< 0.0001
Age of the farmer (by 5 years)	0.98	[0.96–0.99]	0.001
Sex of the farmer (female vs male)	0.84	[0.82–0.86]	< 0.0001
IDM quintile 1 (very wealthy) 2 (wealthy) 3 (medium) 4 (deprived) 5 (very deprived)	Rif. 0.98 1.05 1.95 3.73	[0.91–1.05] [1.02–1.08] [1.02–3.81] [3.54–3.94]	0.585 < 0.0001 0.047 < 0.0001
Ind_013—Employment rate	0.91	[0.86–0.97]	0.003
Ind_018—Cultural demand	0.84	[0.67–0.86]	0.004
Ind_044—Air traffic index	0.98	[0.97–0.99]	< 0.0001
Ind_105—Tourism rate	0.94	[0.92–0.97]	< 0.0001
Ind_135—Micro criminality Index	1.21	[1.11–1.29]	< 0.0001
Ind_141—Hospital emigration	0.72	[0.63–0.83]	< 0.0001
Ind_278—Flood risk population (by 100 inhabitants/km^2^)	1.19	[1.08–1.32]	0.001
Ind_279—Rate of reported thefts	1.02	[1.01–1.03]	< 0.0001
Ind_280—Rate of reported robberies	1.43	[1.41–1.46]	< 0.0001
Ind_281—Homicide rate	1.31	[1.26–1.35]	< 0.0001
