# Supplementary material for: Socio-economic factors as indicators for various animal diseases in Sardinia
Source: PLoS One. 2019 Jun 3;14(6):e0217367. doi: 10.1371/journal.pone.0217367 (PMC6546212; doi:10.1371/journal.pone.0217367)
Supplement: S1 Table — (DOCX) [file pone.0217367.s001.docx]

**S1 Table. List of variable collected by the macro-area of arguments.**

| **VARIABLE COLLECTED** | **VARIABLE DEFINITION** | **MACROAREA OF ARGUMENT** |
| --- | --- | --- |
| N cases | Number of notified cases, by disease | SIMAN |
| ASL | Local Sanitary Agency | BDN |
| Species | Species of animal (sheep and goats, bovine, swine, mixed) | BDN |
| ID farm | The farm code of origin | BDN |
| Type of farm | Typology of farm (intensive, backyard) | BDN |
| N farms | The number of total farms in each municipality, by year | BDN |
| N. animals | The number of total animal (susceptible species) censed in each municipality, by year | BDN |
| Age of the farmer | Years | AgriISTAT |
| Sex of the farmer | Male/Female | AgriISTAT |
| IDM | Material Deprivation Index | ISTAT – Università di Cagliari |
| Ind_007 - Unpolluted coasts for pollution | kilometres of coasts unpolluted over total kilometres of coasts | ISTAT - Environment |
| Ind_009 – Efficiency of water distribution | Water supplied/total amount of water introduced into the municipal distribution networks (percentage) | ISTAT – Water Resources |
| Ind_012 - Unemployment rate | Job seekers 15 years and older/ workforce in the corresponding age group (%) | ISTAT - Work |
| Ind_013 - Employment rate | 15-64 years old employed people/ population in the corresponding age group (%) | ISTAT - Work |
| Ind_014 - Employment rate over 54 years | Employed people 54 years and older/ population in the corresponding age group (%) | ISTAT - Work |
| Ind_015 – Young unemployment rate | Employed people 15-24 years old/ population in the corresponding age group (%) | ISTAT - Work |
| Ind_018 - Cultural demand | Number of visitors to antiquity and art national institutes of the state institute (thousands) | ISTAT – Cultural heritages |
| Ind_024 - Degree of promotion of the cultural offer of state institutions | Paying visitors/ non-paying visitors to state institutes of antiquities and art with paid admission (%) | ISTAT – Cultural heritages |
| Ind_027 - Degree of diffusion of theatrical and musical entertainment | Tickets sold for theatrical and musical activities (number per 100 inhabitants) | ISTAT – Cultural heritages |
| Ind_044 - Air traffic index | Passengers disembarked and boarded by air (number per 100 inhabitants) | ISTAT - Tourism |
| Ind_052 - Separate municipal waste | Urban separate waste/ total urban waste (%) | ISTAT - Garbage |
| Ind_057 - Difference between male and female employment rate | Absolute difference between male and female employment rate in the 15-64 year age-group (%) | ISTAT - Work |
| Ind_060 – Interruption of the electricity service | Frequency of the accidental long interruptions of the electricity service (average number per user) | ISTAT - Energy |
| Ind_080 - Energy produced from renewable sources | GWh of energy produced from renewable sources/ GWh produced in total (%) | ISTAT - Energy |
| Ind_083 - Municipal waste | Kg of urban waste collected/ inhabitant | ISTAT - Garbage |
| Ind_105 - Tourism rate | Days of presence (Italian and foreign) in the complexes of accommodation facilities per inhabitant | ISTAT - Tourism |
| Ind_108 - Participation of the population in the labour market | Labour force aged 15-64 years/ total population aged 15-64 years (%) | ISTAT - Work |
| Ind_120 - Weight of cooperative society | Employees of cooperative companies/ total number of employees (%) | ISTAT – Social capital |
| Ind_135 - Micro criminality index | Crimes linked to petty crime in cities /1.000 inhabitants | ISTAT – Legality and security |
| Ind_141 - Hospital emigration | Hospital emigration to another region for acute ordinary hospitalizations/ total hospitalized persons residing in the region (%) | ISTAT – Health care |
| Ind_142 - Childhood services | Percentage of Municipalities that have activated childcare services (nursery school, micronids or supplementary and innovative services) out of the total number of municipalities in the province | ISTAT - Health care |
| Ind_162 - Funding risk | Decay rate of cash loans (%) | ISTAT - Finance |
| Ind_165 - Tourism in not-summer period | Days of presence (Italians and foreigners) in the complexes of facilities in the non-summer months/inhabitant | ISTAT - Tourism |
| Ind_168 - Ability to export in sectors with dynamic global demand | Share of the value of exports in sectors with dynamic global demand/ total exports (%) | ISTAT - Internationalization |
| Ind_175 - Unemployment rate (male) | Job seekers 15 years and older (male)/ workforce in the corresponding age group (%) | ISTAT - Work |
| Ind_176 - Unemployment rate (female) | Job seekers 15 years and older (female)/ workforce in the corresponding age group (%) | ISTAT - Work |
| Ind_177 - Employment rate (male) | Employed people in the 15-64 years age group (male)/ population in the corresponding age group (%) | ISTAT - Work |
| Ind_178 - Employment rate (female) | Employed people in the 15-64 years age group (female)/ population in the corresponding age group (%) | ISTAT - Work |
| Ind_232 - Percentage of municipal waste disposed of in landfills | Urban waste disposed of in landfill on urban waste produced (percentage) | ISTAT - Garbage |
| Ind_239 - Forests surface | Forest surface (hectares) | ISTAT - Environment |
| Ind_241 - Enrolment gross rate in the business register | Companies registered/ total number of companies registered in the previous year (%) | ISTAT – Business demographics |
| Ind_242 - Enrolment net rate in the business register | Companies registered (minus companies ceased)/ total number of companies registered in the previous year (%) | ISTAT – Business demographics |
| Ind_255 - Forests surface burned by fire | Forest surface destroyed by fire over total forest area (km^2^) | ISTAT - Environment |
| Ind_265 - Air quality monitoring | Equipped with air monitoring stations / 100.000 inhabitants | ISTAT – Cities |
| Ind_278 - Flood risk population | Inhabitants exposed to flood risk (by km^2^) | ISTAT - Environment |
| Ind_279 - Rate of reported thefts | Reported thefts /1.000 inhabitants | ISTAT – Legality and security |
| Ind_280 - Rate of reported robberies | Reported robberies/1.000 inhabitants | ISTAT – Legality and security |
| Ind_281 - Homicides rate | Voluntary homicides/1.000 inhabitants | ISTAT – Legality and security |
| Ind_414 - Taking charge of all users of childcare services | Children between 0-3 years who have used childcare services (nursery or supplementary and innovative services)/ total population aged 0-3 years (%) | ISTAT - Health care |
| Ind_415 - Elderly in social assistance | Elderly treated in social assistance/ total elderly population (65 years and over) (%) | ISTAT - Health care |
| Ind_445 - Index of accessibility to urban nodes | Travel times towards urban and logistics nodes | ISTAT - Tourism |
