# Supplementary material for: Socio-economic factors as indicators for various animal diseases in Sardinia
Source: PLoS One. 2019 Jun 3;14(6):e0217367. doi: 10.1371/journal.pone.0217367 (PMC6546212; doi:10.1371/journal.pone.0217367)
Supplement: S2 Table — (DOCX) [file pone.0217367.s002.docx]

**S2 Table. Italian cases distribution (excluding Sardinia), by study-year (2011-2018) and type of disease.**

| **Year** | **Bluetongue** | **West Nile (human and animals)** | **CE (sheeps)*** | **Contagious Agalactia (sheep and goats)** |
| --- | --- | --- | --- | --- |
| 2011 | 11 | 61 | 88 | 27 |
| 2012 | 13 | 40 | 100 | 13 |
| 2013 | 271 | 133 | 97 | 8 |
| 2014 | 1636 | 118 | 95 | 7 |
| 2015 | 313 | 119 | 90 | 4 |
| 2016 | 1105 | 153 | 93 | 5 |
| 2017 | 175 | 140 | 91 | 1 |
| 2018 | 59 | 404 | 87 | 0 |
| **Total** | **3583** | **1168** | **741** | **65** |

*number of CE cases expressed as number of sheeps found positive during slaughterhouse inspection
