# Supplementary material for: Socio-economic factors as indicators for various animal diseases in Sardinia
Source: PLoS One. 2019 Jun 3;14(6):e0217367. doi: 10.1371/journal.pone.0217367 (PMC6546212; doi:10.1371/journal.pone.0217367)
Supplement: S3 Table — Data are divided by disease (corresponding to different NBRM) and presented as P-value resulting from univariable analysis, and final decision of inclusion (Yes) or exclusion (Not), based on p-value, variance inflation factor (i.e. multicollinearity evaluation), experimental requirements. (DOCX) [file pone.0217367.s003.docx]

**S3 Table. Final decision of inclusion in the final Negative Binomial Regression Models (NBRMs) for each variables collected. Data are divided by disease (corresponding to different NBRM) and presented as P-value resulting from univariable analysis, and final decision of inclusion (Yes) or exclusion (Not), based on p-value, variance inflation factor (i.e. multicollinearity evaluation), experimental requirements.**

| **OUTCOME** | **AFRICAN SWINE FEVER in domestic pigs** | | **AFRICAN SWINE FEVER in wild boar** | | **CONTAGIOUS AGALACTIA** | | **WEST NILE DISEASE** | |
| --- | --- | --- | --- | --- | --- | --- | --- | --- |
| **VARIABLE** | **P-value** | **Final Decision: Inclusion/**  **Exclusion** | **P-value** | **Final Decision: Inclusion/**  **Exclusion** | **P-value** | **Final Decision: Inclusion/**  **Exclusion** | **P-value** | **Final Decision: Inclusion/**  **Exclusion** |
| N. farms | 0.1958* | Yes | 0.3244 | Not | 0.1478* | Yes | 0.7373 | Not |
| N. animals | 0.1066* | Yes | 0.6452 | Not | 0.1336* | Yes | 0.7212 | Not |
| Age of the farmer | 0.1423* | Yes | 0.1981* | Not | 0.1025* | Yes | 0.1839* | Not |
| Sex of the farmer | 0.0855* | Yes | 0.1873* | Not | 0.1854* | Yes | 0.1387* | Not |
| IDM | 0.2004* | Yes | 0.1661* | Not | 0.9485 | Not | 0.1641* | Not |
| Ind_007 - Unpolluted coasts for pollution | 0.3487 | Not | 0.6532 | Not | 0.7009 | Not | 0.6963 | Not |
| Ind_009 – Efficiency of water distribution | 0.8466 | Not | 0.1584* | Not | 0.1846* | Not | 0.1823* | Not |
| Ind_012 - Unemployment rate | 0.6581* | Not | 0.1845* | Not | 0.6501 | Not | 0.6091 | Not |
| Ind_013 - Employment rate | 0.1383* | Yes | 0.1784* | Yes | 0.2003* | Not | 0.1225* | Yes |
| Ind_014 - Employment rate over 54 years | 0.7238 | Not | 0.3375 | Not | 0.2128 | Not | 0.7619 | Not |
| Ind_015 – Young unemployment rate | 0.6544 | Not | 0.4590 | Not | 0.8629 | Not | 0.7940 | Not |
| Ind_018 - Cultural demand | 0.1183* | Yes | 0.1254* | Yes | 0.1022* | Yes | 0.1365* | Yes |
| Ind_024 - Degree of promotion of the cultural offer of state institutions | 0.5699 | Not | 0.2985 | Not | 0.6075 | Not | 0.7518 | Not |
| Ind_027 - Degree of diffusion of theatrical and musical entertainment | 0.2254 | Not | 0.7455 | Not | 0.7032 | Not | 0.4643 | Not |
| Ind_044 - Air traffic index | 0.1314* | Not | 0.6333* | Not | 0.1395* | Not | 0.1527* | Not |
| Ind_052 - Separate municipal waste | 0.1652* | Not | 0.1455* | Yes | 0.1519* | Not | 0.2785 | Not |
| Ind_057 - Difference between male and female employment rate | 0.7316 | Not | 0.4158 | Not | 0.1698* | Not | 0.4484 | Not |
| Ind_060 – Interruption of the electricity service | 0.5183 | Not | 0.3963 | Not | 0.5255 | Not | 0.5408 | Not |
| Ind_080 - Energy produced from renewable sources | 0.2258 | Not | 0.1847* | Yes | 0.6692 | Not | 0.1930* | Yes |
| Ind_083 - Municipal waste | 0.1986* | Not | 0.4641 | Not | 0.1633* | Yes | 0.2344 | Not |
| Ind_105 - Tourism rate | 0.1633* | Not | 0.7663 | Not | 0.5573 | Not | 0.7973 | Not |
| Ind_108 - Participation of the population in the labour market | 0.7892 | Not | 0.3468 | Not | 0.4611 | Not | 0.3402 | Not |
| Ind_120 - Weight of cooperative society | 0.3245 | Not | 0.7843 | Not | 0.3977 | Not | 0.3482 | Not |
| Ind_135 - Micro criminality index | 0.1854* | Yes | 0.1158* | Yes | 0.1452* | Yes | 0.1828* | Not |
| Ind_141 - Hospital emigration | 0.2587 | Not | 0.9851 | Not | 0.5099 | Not | 0.1920* | Not |
| Ind_142 - Childhood services | 0.3347* | Not | 0.8705 | Not | 0.2268 | Not | 0.2220 | Not |
| Ind_162 - Funding risk | 0.5918 | Not | 0.3902 | Not | 0.5341 | Not | 0.4449 | Not |
| Ind_165 - Tourism in not-summer period | 0.1986* | Yes | 0.1566* | Not | 0.1250* | Yes | 0.6363 | Not |
| Ind_168 - Ability to export in sectors with dynamic global demand | 0.5976 | Not | 0.1368* | Not | 0.8340 | Not | 0.7826 | Not |
| Ind_175 - Unemployment rate (male) | 0.1128* | Not | 0.1948* | Not | 0.2087* | Not | 0.2292 | Not |
| Ind_176 - Unemployment rate (female) | 0.1174* | Not | 0.1385* | Not | 0.2000* | Not | 0.2437 | Not |
| Ind_177 - Employment rate (male) | 0.1389* | Not | 0.1733* | Not | 0.1946* | Not | 0.2753 | Not |
| Ind_178 - Employment rate (female) | 0.1156* | Not | 0.1729* | Not | 0.1546* | Not | 0.2977 | Not |
| Ind_232 - Percentage of municipal waste disposed of in landfills | 0.4596 | Not | 0.5809 | Not | 0.5967 | Not | 0.9031 | Not |
| Ind_239 - Forests surface | 0.1616* | Not | 0.1477* | Yes | 0.1900* | Yes | 0.1569* | Yes |
| Ind_241 - Enrolment gross rate in the business register | 0.3966 | Not | 0.8705 | Not | 0.7482 | Not | 0.6417 | Not |
| Ind_242 - Enrolment rate in the business register | 0.6988 | Not | 0.9502 | Not | 0.4320 | Not | 0.5696 | Not |
| Ind_255 - Forests surface burned by fire | 0.1756* | Not | 0.9219 | Not | 0.8740 | Not | 0.9503 | Not |
| Ind_265 - Air quality monitoring | 0.1854* | Not | 0.3600 | Not | 0.4952 | Not | 0.9677 | Not |
| Ind_278 - Flood risk population | 0.1588* | Yes | 0.1865* | Yes | 0.1743* | Yes | 0.1156* | Yes |
| Ind_279 - Rate of reported thefts | 0.1689* | Yes | 0.0986* | Yes | 0.1302* | Yes | 0.5045 | Not |
| Ind_280 - Rate of reported robberies | 0.2001* | Yes | 0.1171* | Not | 0.6313 | Not | 0.6356 | Not |
| Ind_281 - Homicide rate | 0.1853* | Not | 0.9885 | Not | 0.9708 | Not | 0.6929 | Not |
| Ind_414 - Taking charge of all users of childcare services | 0.2435 | Not | 0.5850 | Not | 0.4709 | Not | 0.1443* | Not |
| Ind_415 - Elderly in social assistance | 0.2079* | Not | 0.9488 | Not | 0.1371* | Not | 0.1816* | Not |
| Ind_445 - Index of accessibility to urban nodes | 0.8211 | Not | 0.1568* | Not | 0.2919 | Not | 0.1458* | Not |

| **OUTCOME** | **BLUETONGUE** | | **TRICHINELLOSIS** | | **CISTIC ECHINOCOCCOSIS** | |
| --- | --- | --- | --- | --- | --- | --- |
| **VARIABLE** | **P-value** | **Final Decision: Inclusion/**  **Exclusion** | **P-value** | **Final Decision: Inclusion/**  **Exclusion** | **P-value** | **Final Decision: Inclusion/**  **Exclusion** |
| N. farms | 0.1693* | Not | 0.1943* | Yes | 0.1302* | Yes |
| N. animals | 0.1615* | Yes | 0.1743* | Yes | 0.1918* | Yes |
| Age of the farmer | 0.2541 | Not | 0.1553* | Yes | 0.1754* | Yes |
| Sex of the farmer | 0.5607 | Not | 0.1311* | Yes | 0.1704* | Yes |
| IDM | 0.1371* | Not | 0.1251* | Yes | 0.1205* | Yes |
| Ind_007 - Unpolluted coasts for pollution | 0.1430* | Not | 0.3548 | Not | 0.8917 | Not |
| Ind_009 – Efficiency of water distribution | 0.1999* | Not | 0.1945* | Not | 0.7758 | Not |
| Ind_012 - Unemployment rate | 0.1883* | Yes | 0.1813* | Not | 0.1454* | Not |
| Ind_013 - Employment rate | 0.5135 | Not | 0.1843* | Yes | 0.1526* | Yes |
| Ind_014 - Employment rate over 54 years | 0.7195 | Not | 0.5376 | Not | 0.7336 | Not |
| Ind_015 – Young unemployment rate | 0.8413 | Not | 0.3071 | Not | 0.6326 | Not |
| Ind_018 - Cultural demand | 0.1679* | Yes | 0.1567* | Yes | 0.1253* | Yes |
| Ind_024 - Degree of promotion of the cultural offer of state institutions | 0.8441 | Not | 0.8435 | Not | 0.5942 | Not |
| Ind_027 - Degree of diffusion of theatrical and musical entertainment | 0.8763 | Not | 0.4072 | Not | 0.5201 | Not |
| Ind_044 - Air traffic index | 0.8564 | Not | 0.1293* | Not | 0.1449* | Yes |
| Ind_052 - Separate municipal waste | 0.1891* | Not | 0.1860* | Not | 0.9163 | Not |
| Ind_057 - Difference between male and female employment rate | 0.9710 | Not | 0.7406 | Not | 0.4879 | Not |
| Ind_060 – Interruption of the electricity service | 0.1586* | Not | 0.1663* | Not | 0.6904 | Not |
| Ind_080 - Energy produced from renewable sources | 0.1753* | Not | 0.1467* | Yes | 0.4330 | Not |
| Ind_083 - Municipal waste | 0.1375* | Not | 0.1645* | Not | 0.9878 | Not |
| Ind_105 - Tourism rate | 0.1880* | Yes | 0.1678* | Not | 0.1527* | Yes |
| Ind_108 - Participation of the population in the labour market | 0.7243 | Not | 0.6768 | Not | 0.1747* | Yes |
| Ind_120 - Weight of cooperative society | 0.4048 | Not | 0.4408 | Not | 0.7963 | Not |
| Ind_135 - Micro criminality index | 0.3758 | Not | 0.1904* | Yes | 0.6220 | Yes |
| Ind_141 - Hospital emigration | 0.2870 | Not | 0.2913 | Not | 0.1749* | Yes |
| Ind_142 - Childhood services | 0.2062* | Not | 0.5962 | Not | 0.9061 | Not |
| Ind_162 - Funding risk | 0.4886 | Not | 0.8926 | Not | 0.6382 | Not |
| Ind_165 - Tourism in not-summer period | 0.3044 | Not | 0.1479* | Yes | 0.1796* | Not |
| Ind_168 - Ability to export in sectors with dynamic global demand | 0.8596 | Not | 0.6600 | Not | 0.9034 | Not |
| Ind_175 - Unemployment rate (male) | 0.9292 | Not | 0.1463* | Not | 0.1238* | Not |
| Ind_176 - Unemployment rate (female) | 0.2178 | Not | 0.1423* | Not | 0.1938* | Not |
| Ind_177 - Employment rate (male) | 0.9615 | Not | 0.1509* | Not | 0.1658* | Not |
| Ind_178 - Employment rate (female) | 0.7670 | Not | 0.1997* | Not | 0.1034* | Not |
| Ind_232 - Percentage of municipal waste disposed of in landfills | 0.8410 | Not | 0.9757 | Not | 0.9159 | Not |
| Ind_239 - Forests surface | 0.1676* | Yes | 0.1652* | Not | 0.3811 | Not |
| Ind_241 - Enrolment gross rate in the business register | 0.3681 | Not | 0.2595 | Not | 0.6134 | Not |
| Ind_242 - Enrolment rate in the business register | 0.4806 | Not | 0.4588 | Not | 0.3678 | Not |
| Ind_255 - Forests surface burned by fire | 0.1991* | Not | 0.1808* | Not | 0.8500 | Not |
| Ind_265 - Air quality monitoring | 0.1491* | Yes | 0.1514* | Not | 0.1205* | Not |
| Ind_278 - Flood risk population | 0.1845* | Yes | 0.5239 | Not | 0.1267* | Yes |
| Ind_279 - Rate of reported thefts | 0.6692 | Not | 0.1459* | Yes | 0.1544* | Yes |
| Ind_280 - Rate of reported robberies | 0.3952 | Not | 0.1439* | Not | 0.1819* | Yes |
| Ind_281 - Homicide rate | 0.1919* | Yes | 0.1367* | Yes | 0.1406* | Yes |
| Ind_414 - Taking charge of all users of childcare services | 0.8747 | Not | 0.3152 | Not | 0.6062 | Not |
| Ind_415 - Elderly in social assistance | 0.5504 | Not | 0.9787 | Not | 0.6354 | Not |
| Ind_445 - Index of accessibility to urban nodes | 0.1306* | Not | 0.1932* | Not | 0.7710 | Not |
